# Transformation of Internal Thoracic Structures of *Callobruchus maculatus* (Coleoptera: Bruchidae) from Larva to Adult

**DOI:** 10.3390/insects16030324

**Published:** 2025-03-19

**Authors:** Sipei Liu, Xiaokun Liu, Lijie Zhang, Xieshuang Wang, Xinying Zhang, Le Zong, Wenjie Li, Zhengzhong Huang, Xin Liu, Siqin Ge

**Affiliations:** 1 Key Laboratory of Animal Biodiversity Conservation and Integrated Pest Management, Chinese Academy of Sciences, Beijing 100101, China; spliu@ioz.ac.cn (S.L.); liuxiaokun22@ioz.ac.cn (X.L.); 18801161767@163.com (X.W.); sdauzbxyz@163.com (X.Z.); zongle@ioz.ac.cn (L.Z.); liwenjie@ioz.ac.cn (W.L.); huangzz@ioz.ac.cn (Z.H.); 2 University of Chinese Academy of Sciences, Beijing 100101, China; 3 Science and Technology Research Center of China Customs, Beijing 100101, China; zhanglijie8820@163.com; 4 Department of Medical Research the Ninth Medical Center Chinese PLA General Hospital, Beijing 100101, China

**Keywords:** *Callobruchus maculatus*, 3D reconstruction, metamorphosis, thoracic morphology

## Abstract

*Callobruchus maculatus* is a major quarantine pest of stored legumes in China. As a holometabolous insect, it lives inside the bean it burrows into in both its larval and pupal stages. This study utilized micro-CT and 3D reconstruction to document thoracic morphological transformation during larval and pupal stages. The multi-peak fitting of cranial width was applied to determine larval instars. The results indicate that the first-instar larvae bore into beans using prothoracic muscles and those connecting the head to the mesothorax. The second-instar larva possessed the highest number of thoracic muscles, likely correlating with peak boring activity. The prepupa and the initial pupa exhibited minimal musculature, suggesting larval muscle degradation prior to pupation. Muscles unique to prepupae might homologize with indirect flight muscles in pupae, implying that adult flight capability is determined in the final larval stage. The muscles of both larvae and pupae undergo changes in attachment site, shape and curvature throughout development. At the same time, changes also occur in the larval cuticle and pupal endoskeleton. During the larval stage, muscle growth and degradation occur simultaneously, influencing muscle volume. In the pupal stage, the progressive increase in both absolute and relative thoracic muscle volumes prepare the weevil for movement after emergence. Meanwhile, the other thoracic organs, including the gut, air sacs and nerves, also change during development.

## Simple Summary

The growth of *Callobruchus maculatus*, an important quarantine pest in China, progresses through four stages: egg, larva, pupa and adult. During the larval and pupal stages, this weevil sustains itself by boring into beans. This study used advanced morphological imaging techniques to create 3D reconstructions of the internal thoracic structures of larvae and pupae of *C. maculatus*. To classify larval instars, multi-peak fitting was applied based on the measurements of cranial width. The first-instar larvae use prothoracic muscles and those connecting the head to the mesothorax to bore into beans. The second-instar larva has the most thoracic muscles and likely possesses the greatest boring ability. The prepupa and the initial-pupal-stage weevil have the fewest muscles, indicating that larval muscles might degrade before pupation. Some muscles in the prepupa may be homologous to the indirect flight muscles that only appear in the flight form during the initial pupal stage, suggesting that the determination of adult form occurs in the final larval stage. Throughout development, the muscles, larval cuticle and pupal endoskeleton undergo changes in attachment, shape and size. During the larval stage, both muscle growth and degradation influence muscle volumes, while in the pupal stage, the progressive increase in both absolute and relative thoracic muscle volumes prepares the insect for movement after emergence. Simultaneously, the gut, air sacs and nerves also change as the larva and pupa grow and develop.

## 1. Introduction

**Figure 1:**
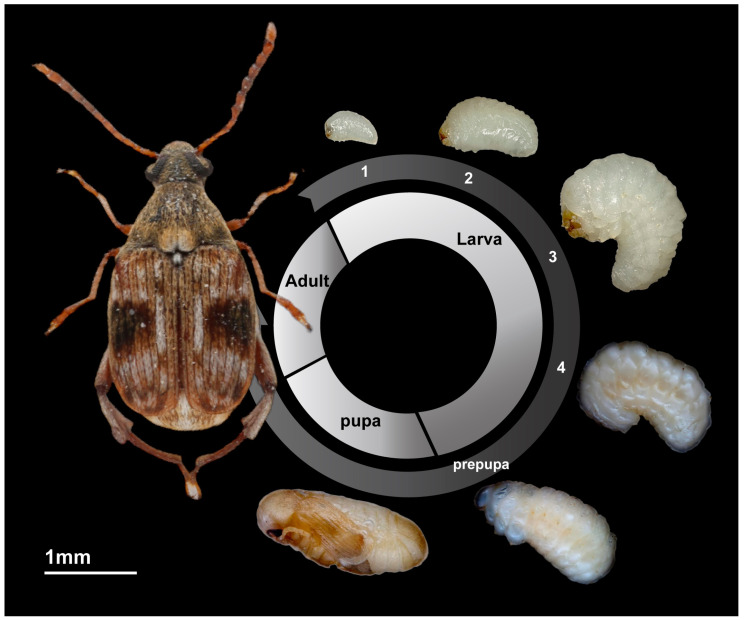
First- to fourth-instar larvae, pupa and adult of *Callosobruchus maculatus*.

*Callobruchus maculatus* is a globally significant pest of stored legumes, including mung beans, cowpeas, chickpeas, soybeans, red beans and so on [1]. Introduced to China in the 1960s, it is found in parts of the Zhejiang and Taiwan provinces [2]. As a holometabolous insect, *C. maculatus* experiences four developmental stages: egg, larva, pupa and adult (Figure 1). The female weevil lays her eggs on the surface of the bean. After hatching, the first-instar larvae chew through the bean skin beneath the eggs and bore into the bean. They remain inside until they are ready to emerge. In cases of severe infestation, more than ten weevils could inhabit a single bean simultaneously. The adult weevil then bites through the bean skin from the inside, creating a round “window” through which it crawls out. Adults exhibit polyphenism: flight-capable forms disperse but are less fecund, while non-flying forms are reproductively active [3,4].

Holometabolous insects undergo dramatic internal reconstruction during metamorphosis [5]. With the advent of advanced morphological techniques, their internal structural changes during development can now be recorded with high accuracy. The transformations of one or more of the muscles, tracheae, guts, nerves, malpighian tubules or fat bodies in *Calliphora vicina*, *Vanessa cardui*, *Oestrus ovis*, *Megachile rotundata*, *Chrysopa pallens* and *Drosophila melanogaster* are documented based on micro-CT and 3D reconstruction [6,7,8,9,10,11,12]. In studies of Coleopteran insects, the effects of miniaturization were examined through the anatomical details of larvae, pupae, adults of *Sericoderus lateralis* (Corylophidae) [13]. A comparison of the head anatomical structures across a third-instar larva, fourth-day pupa and adult of *Chrysomela populi* (Chrysomelidae) revealed that the pupa demonstrated a combination of adult and larval characteristics [14]. Changes in the tracheal structure during the development of *Tenebrio molitor* (Tenebrionidae) were quantitatively recorded [15]. A comparison of the gut, nerve and reproductive systems during pupal development between *T. molitor* and *Tribolium castaneum* suggests species-specific adaptations of the pro-imaginal stages to environmental conditions [16]. The nerve, digestive and excretion systems, as well as body muscles, were documented during the transformation process of *Harmonia axyridis* (Coccinellidae) [17].

In the present study, we used micro-CT and 3D reconstruction techniques to comprehensively document changes in the thoracic anatomical structure throughout the larval and pupal development of *Callobruchus maculatus*. This period covers the entire duration of the infestation of beans by *C. maculatus*. The larvae live inside beans, making their molting impossible to observe directly. Therefore, we applied multi-peak fitting to estimate their instar based on Dyar’s law, which states that increases in highly sclerotized body parts are predictable and regular, following a relatively constant factor [18]. In the coleopteran larval instar classification, the widths of the head capsule, cranium and mandible are commonly used as measurement indicators [19,20,21,22]. Since the head of the *C. maculatus* larva is retracted into the prothorax, we selected the cranial width, namely the distance between the antennal fossae, as a measurement indicator. Through the transformation of thoracic morphology in the larvae and pupae of *C. maculatus*, we hope to achieve a better understanding of the developmental progress of this storage pest, contributing to more effective integrated pest management.

## 2. Materials and Methods

### 2.1. Specimen Rearing

The *Callobruchus maculatus* specimens used in this study were from Science and Technology Research Center of China Customs (Beijing, China), where a batch of cowpeas from Nigeria was intercepted. The weevils were raised in Petri dishes containing cowpea on moist filter paper, which were placed in a laboratory culture maintained at a constant temperature of 28 °C and 75% relative humidity under a 16 h–8 h light–dark photoperiod [23].

### 2.2. Larval Specimen Collection

**Figure 2:**
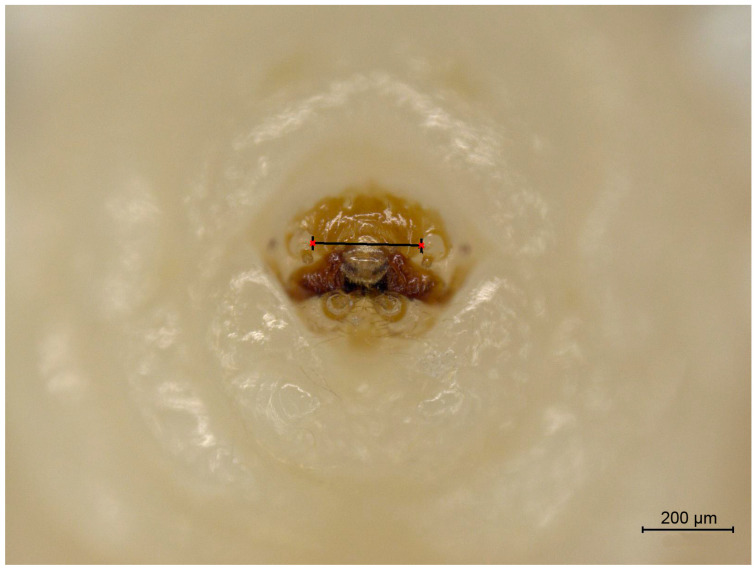
Cranial width of larva for instar classification.

From the appearance of oval white eggshells in the Petri dishes, batches of cowpeas were soaked in 100% ethanol every three days, with larvae collected until a substantial number of pupae emerged in the cowpeas. A total of 226 larvae were collected. We photographed the front view of the head of each larval specimen and measured the cranial width from the images (Figure 2; Supplementary Material S1). The peak fitting module of Origin 8.1 (OriginLab, Northampton, MA, USA) was used to perform the multi-peak fitting of larva cranial width based on a Gaussian function, aiming to maximize R^2^ to as close to 1 as possible. The solve function of Matlab R2014a (MathWorks, Natick, MA, USA) was used to compute the intersection point of two adjacent Gaussian functions, representing the boundary value of front width for two sequential instars, as well as the coefficient of variation and misclassification probability.

Based on the multi-peak fitting results, one larva from each instar was selected and preserved in larval fixative. The cranial width of fourth-instar larvae and prepupae shows no statistically significant difference. The body of the fourth-instar larva is bent dorsad, while the prepupae have a straight body, allowing them to be directly distinguished by observation. The formula of larval fixative is as follows: acetic acid/distilled water/40% formaldehyde solution/96% ethanol = 4:30:6:15.

### 2.3. Pupal Specimen Collection

Some larvae were collected from cowpea and reared in Petri dishes filled with quartz sand and soybean powder. The pupation and eclosion processes were documented using a mobile phone (HUAWEI P20 Pro, Shenzhen, China). The pupal stage of *C. maculatus* lasts a total of four days. Based on the recorded development process, one specimen was selected at each stage—initial, middle, late pupa and adult—and fixed in 75% ethanol for over one week.

### 2.4. Micro-CT and 3D Reconstruction

The larval specimens needed to be stained with iodine solution for 48 h and rinsed 1–2 times with 75% ethanol. The pupal and adult specimens did not need to be stained. All the specimens were dehydrated in an ascending ethanol series (75%–80%–85%–90%–95%–100%) and dried at the critical point (Leica EM CPD300, IOZCAS, Beijing, China). During the scanning process using micro-CT (Zeiss microXCT-400, IOZCAS, Beijing, China), the specimen was pasted on the base of a pipette tip, with its tip end securely fixed onto the specimen holder of the micro-CT apparatus. The thoracic part of each specimen was reconstructed three-dimensionally based on a micro-CT image stack using Amira 6.0 (Thermo Fisher Scientific, Waltham, MA, USA). The parameters of the micro-CT scanning and 3D reconstructions used here are listed in Table 1. The measurement of muscle volume was conducted using the “Material Statistics” module in Amira. Segmented structures were exported as stacks of tiff files into VG Studio Max 3.0 (Volume Graphics, Heidelberg, Germany) for volume rendering. The final images were edited using Adobe Photoshop 2017 (Adobe Inc., Mountain View, CA, USA) and Adobe Illustrator 2017 (Adobe Inc., Mountain View, CA, USA) for layout and the addition of labels.

### 2.5. Terminology and Abbreviations

In the anatomical study of larvae, we established a naming system for muscles based on their attachment positions on the cuticles. In the study of pupae and adults, the terminology for the endoskeleton follows the work of Ruan et al. [24]; for muscles, the work of Friedrich and Beutel is followed [25].

## 3. Results

### 3.1. Larval Development

#### 3.1.1. Multiple-Peak Fit

**Figure 3:**
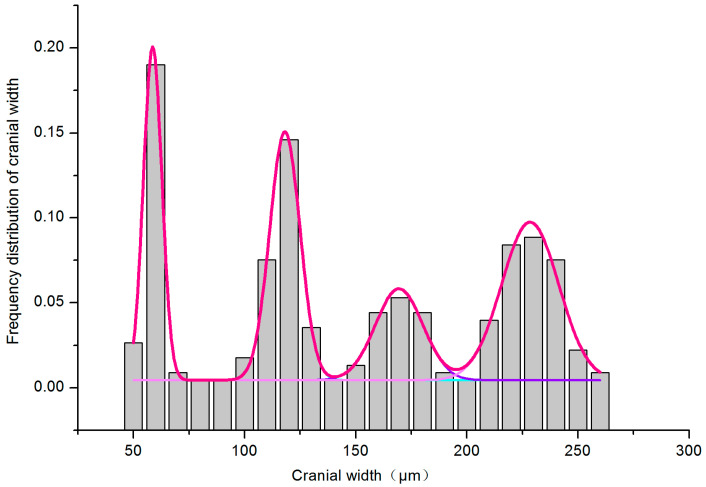
Multi-peak fitting of cranial width. The deep pink line represents the outcome of the multimodal fitting, while the other colors depict the Gaussian function distributions from the original fitting for each instar.

After fitting with the Gaussian function, four significant peaks are observed (Figure 3). The R^2^ value is 0.98, close to 1, with the coefficient of variation for each instar being less than 1 and the misclassification probability at each instar being below 5%, indicating a high-quality fitting result (Table 2).

#### 3.1.2. Cuticles

In the larva of *C. maculatus*, only the head, which is retracted into the prothorax, is sclerotized. The thorax of the larva is covered with a delicate soft cuticle. Each thoracic segment has a pair of ventral legs, except in the first-instar larva. In the prepupa, the pupal cuticle is already present on the inner side of the larval cuticle, indicating that larval–pupal apolysis is basically completed. We broadly divided each thoracic segment into dorsal, lateral and ventral regions. The length, width and height of the thorax were measured at their maximum points (Table 3).

#### 3.1.3. Muscles

**Figure 4:**
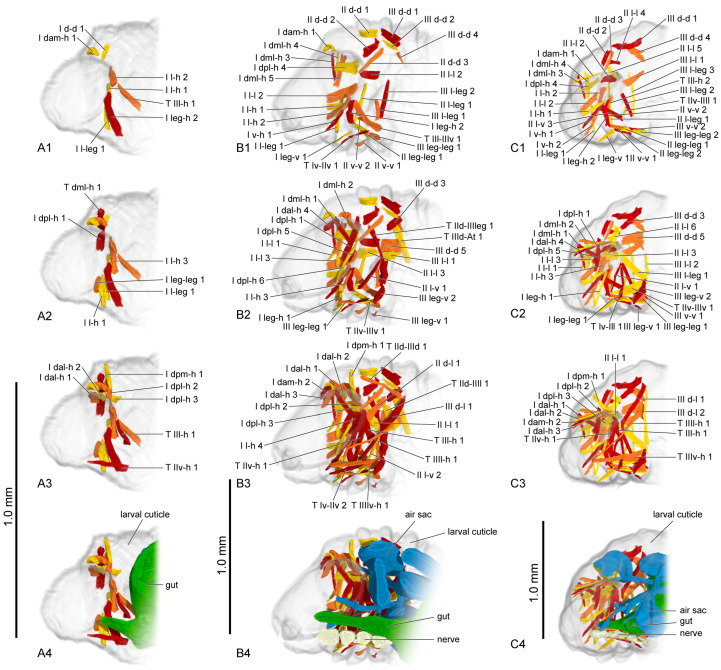
Thoracic musculature and other organs of larval *Callosobruchus maculatus* in sagittal section. (**A1**–**A4**) first instar; (**B1**–**B4**) second instar; (**C1**–**C4**) third instar. The numbers 1–4 indicate a gradual movement from the lateral position of the thorax to the proximal position.

**Figure 5:**
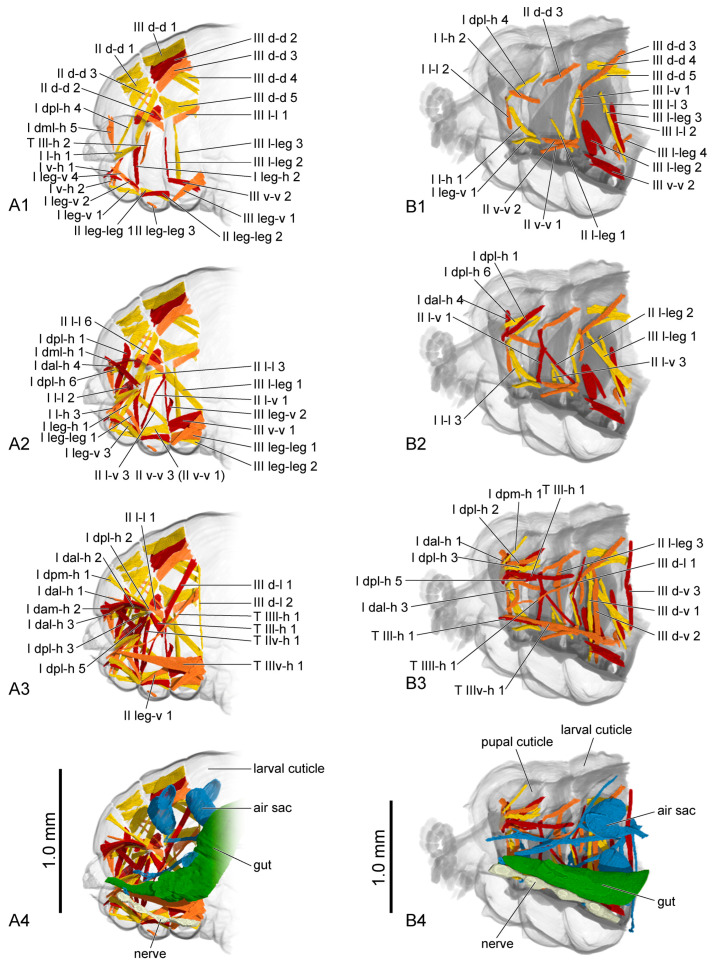
Thoracic musculature and other organs of larval *Callosobruchus maculatus* in sagittal section. (**A1**–**A4**) fourth instar; (**B1**–**B4**) prepupa. The numbers 1–4 indicate a gradual movement from the lateral position of the thorax to the proximal position. II v-v 1 is hidden by II v-v 3 in **A2**.

**Figure 6:**
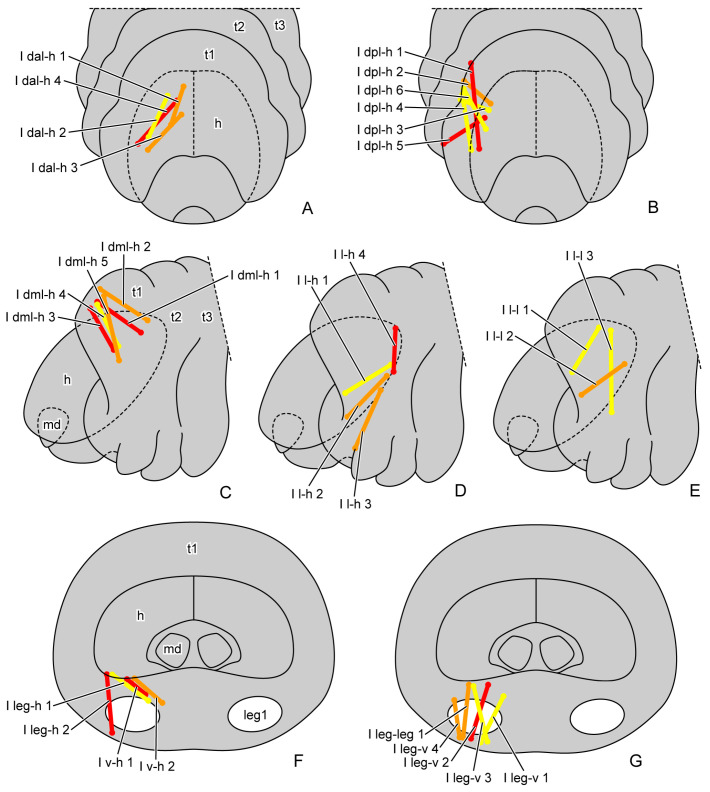
General locations of prothoracic muscles of larval *Callosobruchus maculatus*. (**A**,**B**) front view; (**C**–**E**) lateral view from outside; (**F**,**G**) rear view from inside. Abbreviations: h—head; md—mandible; leg1—proleg; t1/2/3—pro-/meso-/metathorax.

**Figure 7:**
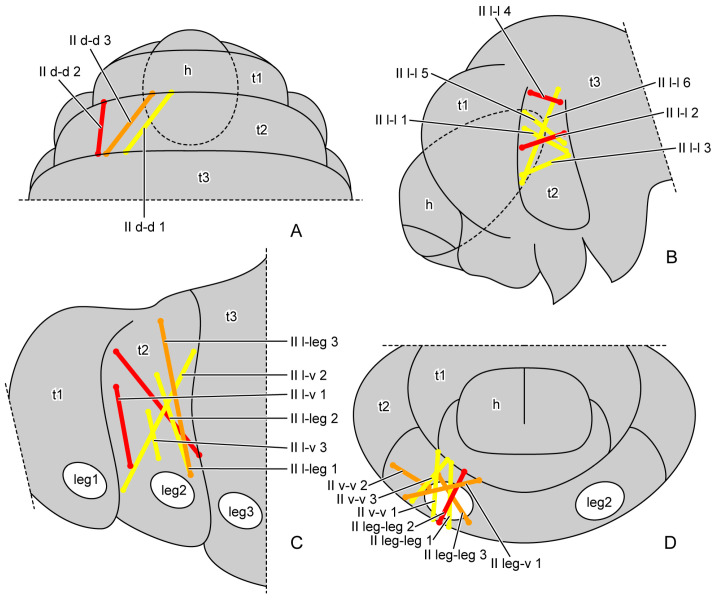
General locations of mesothoracic muscles of larval *Callosobruchus maculatus*. (**A**) dorsal view from outside; (**B**,**C**) lateral view from outside; (**D**) rear view from inside. Abbreviations: h—head; leg1/2/3—pro-/mid-/hindleg; t1/2/3—pro-/meso-/metathorax.

**Figure 8:**
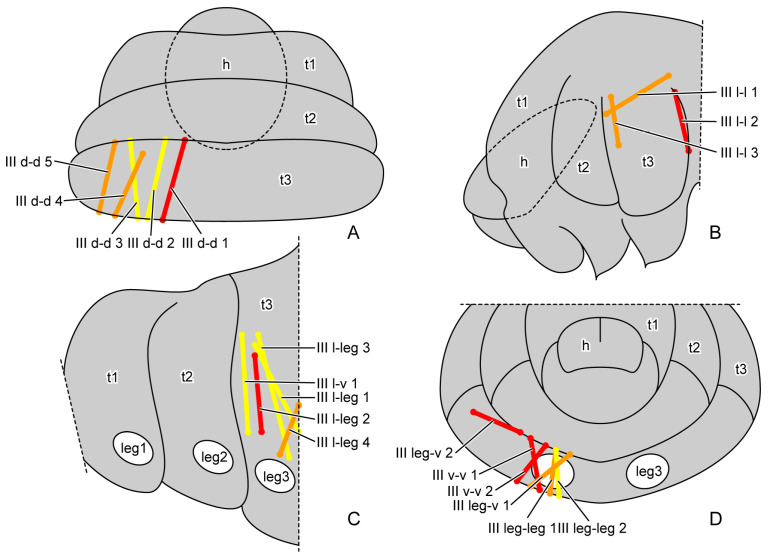
General locations of metathoracic muscles of larval *Callosobruchus maculatus*. (**A**) dorsal view from outside; (**B**,**C**) lateral view from outside; (**D**) rear view from inside. Abbreviations: h—head; leg1–3—pro-/mid-/hindleg; t1–3—pro-/meso-/metathorax.

The muscles were named according to their attachment sites on the inner surface of the thoracic cuticle (Table 4). By combining the muscles from all larval instars, we identified a total of 98 muscles, including 36 in the prothorax, 23 in the mesothorax, 24 in the metathorax and 15 intersegmental muscles. From the first instar to the prepupa, the number of muscles present in the larval thorax at each stage is 20, 68, 69, 62 and 42, respectively (Figure 4 (A1–A3,B1–B3,C1–C3); Figure 5 (A1–A3,B1–B3)). The presence or absence of muscles in each developmental stage was recorded (Table 5). Each muscle at each stage during larval development is described in detail (Supplementary Material S2). The origin, insertion and shape of the muscle at its earliest appearance were recorded. Any changes in subsequent stages were recorded again, while unchanged muscles were not re-recorded. The changes in the muscles in development were also noted. We also measured the volume of each muscle (Supplementary Material S3). The absolute volume was calculated based on the scale bars in the micro-CT results. For the relative volume, the muscular absolute volume was compared with the product of thoracic length, width and height. In addition to 3D reconstruction, we also hand-drew the general locations where certain muscle groups attach to the thoracic cuticle, combining data from all instars (Figure 6, Figure 7 and Figure 8).

#### 3.1.4. Digestive System, Respiratory System and Nervous System

Similarly to the muscles, the other organs are documented at their earliest appearance, with any changes occurring during subsequent development being described as well.

First instar (Figure 4(A4)): The ventral area of the prothorax, the postero-ventral area of the mesothorax, and the metathorax are occupied by a large gut. Only a small anterior portion of the air sac is present in the lateral area of the metathorax. The nervous system cannot be observed.

Second instar (Figure 4(B4)): The narrow anterior part of the gut becomes longer and occupies the ventral area of the thorax. More air sacs are present in the dorsal and median areas of the meso- and metathorax. The ventral nerve cord beneath the gut has four distinct ganglia.

Third instar (Figure 4(C4)): The anterior part of gut gradually enlarges posterad and occupies the postero-ventral area of the thorax. The air sacs and nervous system are nearly the same as in the second instar.

Fourth instar (Figure 5(A4)): The digestive system is nearly the same as in the last stage. The air sacs in the thorax are reduced. The distance between nervous ganglia increases.

Prepupa (Figure 5(B4)): The gut becomes straight and occupies the ventral area of the thorax. The air sacs and the nerve cord are nearly the same as in the fourth instar.

### 3.2. Pupal Development

#### 3.2.1. Skeletons

**Figure 9:**
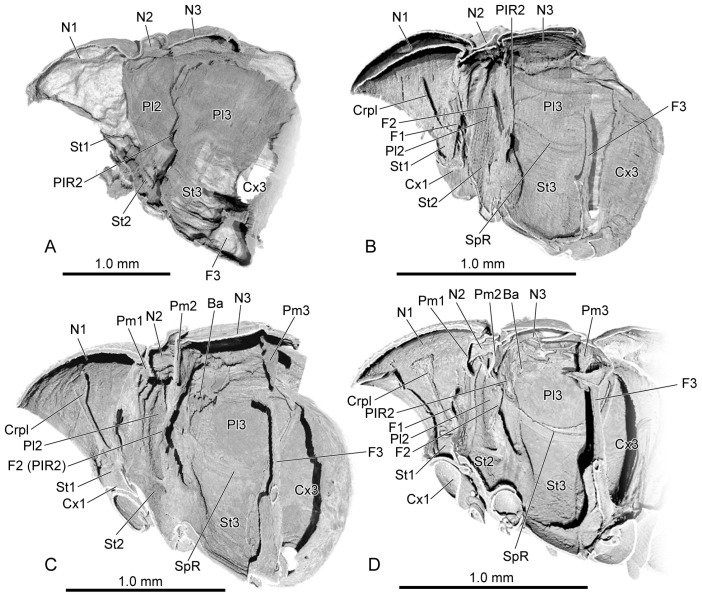
The endoskeleton of pupal and adult *Callosobruchus maculatus*. (**A**) initial pupal stage; (**B**) middle pupal stage; (**C**) late pupal stage; (**D**) adult. Abbreviations: Ba—basalar sclerite; Crpl—cryptopleuron; Cx1/3—pro-/metacoxal; F1/2/3—pro-/meso-/metathoracic furca; N1/2/3—pro-/meso-/metanotum; Pl2/3—meso-/metapleuron; PlR2—mesopleural ridge; Pm1/2/3—pro-/meso-/metaphragma; SpR—sternopleural ridge; St1/2/3—pro-/meso-/metasternum.

Initial pupal stage (Figure 9A): In the dorsal view, the length ratio of the pro-, meso- and metathorax is 20:4:13. The lateral area of the prothorax is long and roughly triangular, narrowing postero-ventrad. A postero-dorsad-bent ridge demarcates the dorsal pronotum (N1) from the ventral prosternum (St1). A horizonal ridge separates the mesonotum (N2) from the anterior pronotum. The ridge between the pronotum and the mesopleuron (Pl2) is anterad-bent. The mesopleuron is narrow, and the mesopleural ridge (PlR2) extends along its posterior margin. The mesosternum is separated from the dorsal mesopleuron by the mesocoxal rim. Another horizonal ridge delimits the metanotum (N3) from the anterior mesonotum. The middle depression of the metanotum on the exterior forms a shallow bulge on the inner side. The large metapleuron (Pl3) is irregularly trapezoid. The large elliptic metacoxa (Cx3) connects to the posterior margin of the metapleuron and metasternum (St3). The raised basal part of the metafurca (F3) is positioned along the posterior section of the metasternal midline.

Middle pupal stage (Figure 9B): In the dorsal view, the length ratio of the pro-, meso- and metathorax is 16:5:9. The slender and curved cryptopleuron (Crpl), with its enlarged base, is situated between the antero-ventral margin of the pronotum (antero-proximal margin of hypomeron) and the antero-lateral margin of the procoxal rim (Cx1). It extends dorsad, near the dorso-lateral margin of the pronotum, with its apex tapering. The prothoracic furca (F1) is located in the postero-median margin of the prosternum. The prothoracic furcal arm extends dorso-laterad, with a slightly enlarged apex. In the mesothorax, the enlarged base of the mesofurca (F2) is split along the midline and located in the posterior area of mesosternum, beneath the mesopleural ridge. The slender mesofurcal arm firstly extends dorsad, and then dorso-laterad at the lower third, with the tip nearly reaching the dorsal area of the mesopleuron. In the metathorax, the borders of the middle bulge on the metanotum are distinct. The ventrad-bent sternopleural ridge (SpR, sternopleural suture on the exoskeleton) demarcates the dorsal metapleuron and ventral metasternum. Both metafurcal arms (F3) are connected with the inverted triangular sclerotized bridge in the lower fourth, with the slender metafurcal arm extending dorsad.

Late pupal stage (Figure 9C): In the dorsal view, the length ratio of the pro-, meso- and metathorax is 15:4:11. The expanded dorsal terminal of the crytopleuron attaches to the dorso-median area of the lateral side of the pronotum (hypomeron). The narrow pro-, meso- and metaphragmas (1/2/3Pm), respectively, expand ventrad along the posterior margin of the pro-, meso- and metanotum. The laterad-bent metabasalare (Ba) is located in the antero-dorsal area of the metapleuron. The basal part of the metafurca, where both metafurcal arms converge, narrows into a stripe. The enlarged apex of each metafurcal arm is bent postero-dorsad.

Adult (Figure 9D): In the dorsal view, the length ratio of the pro-, meso- and metathorax is 13:2:9. The apex of the metafurcal arm expands to form a tray-like structure.

The length, width and height of the thorax were measured at their maximum points (Table 6).

#### 3.2.2. Muscles

**Figure 10:**
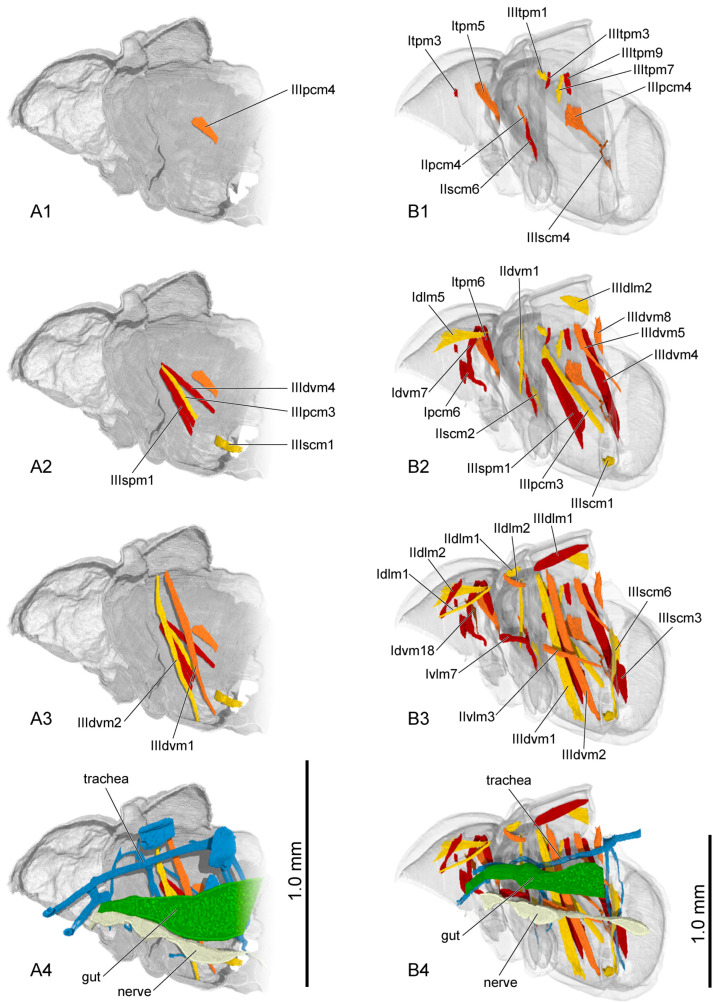
Thoracic musculature and other organs of pupal *Callosobruchus maculatus* in sagittal section. (**A1**–**A4**) initial pupal stage; (**B1**–**B4**) middle pupal stage. The numbers 1–4 indicate a gradual movement from the lateral position of the thorax to the proximal position.

**Figure 11:**
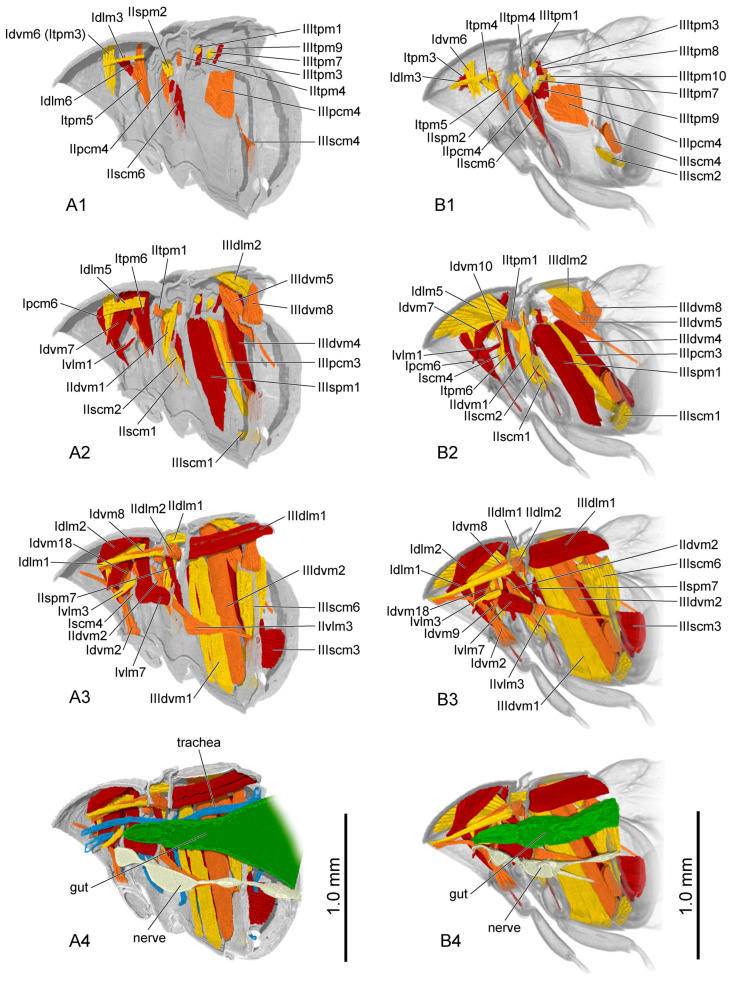
Thoracic musculature and other organs of pupal *Callosobruchus maculatus* in sagittal section. (**A1**–**A4**) late pupal stage; (**B1**–**B4**) adult. The numbers 1–4 indicate a gradual movement from the lateral position of the thorax to the proximal position. Itpm3 is hidden by Idvm6 in (**A1**).

The muscles in the pupal stage were described in the same manner as those in the larval stage (Figure 10 (A1–A3,B1–B3); Figure 11 (A1–A3,B1–B3)). The presence or absence of muscles in each developmental stage was recorded (Table 7). Each muscle at each stage during pupal development is described in detail, including its length and width (Supplementary Materials S4 and S5).

#### 3.2.3. Digestive System, Respiratory System and Nervous System

The other organs in the pupal stage were described in the same manner as those in the larval stage.

Initial pupal stage (Figure 10(A4)): The gut, which gradually thickens from anterior to posterior, is positioned in the ventral area of the thorax. A few oval-shaped air sacs are attached to the tracheae. The nervous cord is composed of three ganglia connected in sequence.

Middle pupal stage (Figure 10(B4)): The gut becomes narrower. The tracheae contain no air sacs. The anterior half of the third nervous ganglion is distinctly tapered.

Late pupal stage (Figure 11(A4)): The gut gradually thickens from anterior to posterior. The tracheae are nearly the same as in the previous stage. The anterior part of the third nervous ganglion slightly broadens.

Adult (Figure 11(B4)): The gut becomes narrower. A slender process extends postero-ventrad from the posterior part of the second nervous ganglion to the metacoxa.

## 4. Discussion

### 4.1. Skeleto-Muscular Transformations During Larval Development

Nearly all muscles present in the first-instar larva are concentrated in the prothorax. Among them, the muscles I dml-h 1, I dpm-h 1 and I l-h 1 have only one end attached to the prothoracic cuticle, while the other end extends toward the head without a direct connection. These muscles might be stabilized by other tissues in the body cavity that cannot be imaged by micro-CT. The other three muscles, T IIl-h 1 and 2 and T IIv-h 1, extend from the mesothorax to the head. The thoracic ventral legs are absent in the first-instar larva. Muscles such as I l-leg 1 and I leg-h 1 and 2, which are attached to the basal rim of the proleg in the other instars, are instead attached to the ventral region of the prothorax in the first-instar larva. After hatching from the egg, the larva begins to burrow into the cowpea for food and shelter. When the larva first begins boring, it relies on the muscles in its prothorax to wriggle and maneuver its head, without the assistance of its legs. In addition, the muscles connected to the body wall at only one end, with the other end unattached, cannot generate force. The muscles in the lateral and ventral regions of the mesothorax, which connect to the head, provide greater torque to control head movement.

The second-instar larva possesses the highest number of thoracic muscles and legs in the ventral region of the thorax. Therefore, we speculate that the second-instar larva might exhibit the strongest boring activity. As development progresses, the number of thoracic muscles gradually decreases from the second instar to the prepupa. This indicates a decline in larval activity and a gradual transition to the pupal stage, probably suggesting that the muscles are entirely destroyed by histolysis during metamorphosis, as observed in *Drosophila* [26,27]. This also implies that muscles appearing during specific developmental periods could serve distinct functions. The muscles II l-leg 2 and 3, III d-v 1–3, III l-l 3, III l-v 1 and III l-leg 4 are present only in the prepupa, a stage after larval–pupal apolysis has occurred. These muscles might be part of the pupal musculature. Due to the limited resolution of micro-CT, some larval muscles might not be reconstructed in 3D. This might be especially true for muscles that are absent during the middle stages of development but present in both the early and late stages, such as I dml-h 5, I dpl-h 6, I l-h 2, I l-l 3, II d-d 1, II l-leg 1, II v-v 2, T IIl-h 2, III d-d 2 and III l-l 2.

Except for a few muscles that appear only in one stage, nearly all other muscles undergo morphological changes during development. The original and insertional sites on the thoracic cuticle of many muscles shift during larval development. Most muscles change in their planar geometry, to forms such as a triangle, parallelogram, trapezoid, etc., as evidenced by alterations in width at their original and insertional ends. Otherwise, most muscles change in curvature, transitioning between straight, curved or bent states, along with variations in their bending direction. The absolute muscle volumes generally exhibit a gradual increase with larval age, though many muscles shrink in the prepupa. In contrast, the relative muscle volumes tend to decrease over time. This pattern likely results from the simultaneous processes of muscle growth and histolysis in the larvae, with muscle degradation becoming predominant during the prepupal stage. It should be noted that the results of measurements are influenced by factors such as the quality of 3D reconstruction and irregular thoracic shape.

### 4.2. Skeleto-Muscular Transformations During Pupal Development

The adults of *Callosobruchus maculatus* have two morphological forms: normal and flight [3]. The normal form exhibits fertility but lacks the ability to fly, whereas the flight form exhibits the opposite. A morphological comparison of the thoracic structures revealed that some muscles are absent in the normal form but present in the flight form [4]. What we studied was the pupal development of the flight form, as specimens at all developmental stages have the muscles IIIdvm1 and 2, IIIspm1 and IIIpcm4, which are absent in the normal form but present in the flight form. Otherwise, for muscles that appear only in the prepupa, based on their attachment positions on the thoracic cuticle, III d-v 1–3, III l-v 1, III l-l 3 and III l-leg 4 might be homologous, respectively, to IIIdvm1, 2 and 4, IIIspm1, and IIIpcm3 and 4 in the initial pupal stage (Table 8). These muscles likely provide indirect power for the flight of the adult by stretching in the vertical direction, which might suggest that flight muscles appear first to provide enough time for the preparation of muscular fibers containing sufficient mitochondria [12]. Indirect flight muscles have also been observed during the prepupal stage of *Harmonia axyridis* [17]. Therefore, it can be concluded that the specimens of the prepupa also belong to the flight form, with the adult form being determined before the larvae complete larval–pupal apolysis. In total, we identified 55 thoracic muscles across all stages of pupal development, with only 7 muscles found in the initial pupal stage. This further suggests that the larval muscles are completely destroyed during pupation, with new muscles developing de novo from remaining undifferentiated embryonic cells throughout the larval period, starting in the final larval stage [26,27].

Some endoskeletal structures appear in the middle pupal stage, which contains the crytopleuron, furcal arms and sternopleural ridge on the metapleuron. In the late pupal stage, the phragmas are suspended along the posterior margins of each notum. The metabasalare is present in the antero-dorsal area of the metapleuron. The crytopleuron reaches full development by the late pupal stage, whereas the metafurca reaches full development by the adult stage. Changes in the endoskeleton during development influence both the attachment sites of the muscles and the timing of their appearance. The muscle Itpm3, which connects to the crytopleural apex, is absent until the middle pupal stage, when the crytopleuron appears. Almost all muscles connected to the furca appear in the middle pupal stage or later, except for IIIscm1. IIIscm1 in the initial pupal stage attaches to the metafurcal basal part. The muscles Idlm1 and 5, IIdlm1 and 2, IIIdlm2, IIIdvm8 and IIItpm1 attach to the intersegmental ridge in the middle pupal stage and later shift their attachment to the phragma in the late pupal stage. IIdlm1 gradually extends anterad in the middle pupal stage and connects to the prophragma in the late pupal stage. Idlm3 and 6, Idvm8 and 10, IIdvm2 and IItpm1 are absent until the late pupal stage or even later, when the phragmas appear. The original sites of IIItpm7, IIIspm1 and IIIpcm3 are positioned in the antero-dorsal or antero-median area of the metapleuron before the late pupal stage. IIItpm3 extends antero-ventrad in the middle pupal stage and attaches to the metabasalare in the late pupal stage.

During pupal development, almost all muscles follow the pattern of developing from absence to presence, except for the muscle Idlm6. Similarly to the absence of Ivlm7 of *Drosophila melanogaster* after eclosion, the loss of Idlm6 might also result from increased scalarization in the adult [12]. The muscles Idvm18, Ipcm6, Iscm4, IIdlm1, IIscm6 and IIItpm3 demonstrate that during muscular formation, one end is connected to the endoskeleton, while the other free end is suspended in the body cavity as shown in *Chrysopa pallens* [11] and *D. melanogaster* [12], possibly anchored by the other tissues [28]. Another mode of muscular extension is also observed in *Callosobruchus maculatus*: the original end of IIIpcm4 is connected to the postero-median area of the metapleuron in the initial pupal stage, and gradually extends along the metapleuron to the antero-dorsal area. Similarly to larval muscles, muscles during pupal development also undergo changes in planar geometry and curvature. Both absolute and relative muscle volumes generally increase during pupal development, preparing the flight-capable *C. maculatus* for extensive movement after emergence.

### 4.3. Transformation of Other Organs During Larval and Pupal Stages

The process of larvae boring into cowpeas is one of continuous feeding, which is related to the first-instar larva having a significantly large gut. However, it is currently difficult to explain why the gut thickens in the late pupal stage. Similarly, the volume of the digestive tract also increases during the late pupal stage compared to earlier pupal stages [17]. The second-instar larva has the most muscle growth and probably the most intense boring activity, requiring more air sacs to supply enough oxygen to support its metabolism. As the larvae pupate, their activity decreases, which might lead to a reduction in oxygen consumption. Consequently, the air sacs also decrease in number or even disappear. By contrast, the respiratory system of *Tenebrio molitor* lacks an air sac structure in both the larval and pupal stages [15]. In the adult, there is a nervous process extending toward the hindleg, which is associated with the activity of the adult burrowing out of the cowpea.

## Figures and Tables

**Table 1:** Parameters of micro-CT scanning and 3D reconstruction. “L1–4”, respectively, represents first- to fourth-instar larvae. “PP” represents the prepupa. “PI”, “PM” and “PL”, respectively, represent the initial, middle and late pupal stages. “A” represents the adult.

Stages	Parameters					
	Voltage (kV)	Current (μA)	Pixel Size (μm)	Voxel Size (μm^3^)	Image Size	Optical Magnification
L1	60	133	0.8456	0.6046	1003 × 1003	9.8225: “10x”
L2			1.5118	3.4552	1012 × 1012	3.9687: “4x”
L3			2.0158	8.1911	1012 × 1012	3.9687: “4x”
L4			2.0661	8.8197	992 × 1005	3.9687: “4x”
PP			2.0920	9.1555	980 × 1003	9.8225: “10x”
PI			1.9607	7.5376	980 × 1003	9.8225: “10x”
PM			1.9685	7.6279	972 × 1003	9.8225: “10x”
PL			1.9607	7.5376	980 × 1003	9.8225: “10x”
A			2.2522	11.4240	988 × 1012	3.9687: “4x”

**Table 2:** Variable values based on the multiple-peak fitting of the cranial width for all instars. “L1–4”, respectively, represents first- to fourth-instar larvae.

Larval Instar	Sample Size	Mean ± SD (µm)	Range (µm)	Coefficient of Variation	Misclassification Probability		
					I as I − 1	I as I + 1	Total
L1	51	58.86 ± 3.76	51.35–74.05	0.0638	-	0.0000	0.0000
L2	64	115.98 ± 9.04	83.92–134.88	0.0779	0.0000	0.0021	0.0021
L3	38	169.57 ± 11.83	140.57–193.91	0.0697	0.0036	0.0124	0.0160
L4 and prepupa	73	229.15 ± 13.01	196.71–264.06	0.0568	0.0022	-	0.0022

**Table 3:** Thoracic length, width and height (µm) of the larvae. “L1–4”, respectively, represent first- to fourth-instar larvae.

	L1	L2	L3	L4	Prepupa
Length	204.75	495.55	781.08	1060.61	1154.03
Width	334.09	819.70	1194.10	1528.95	1523.12
Height	328.15	706.39	898.46	1331.37	1435.07

**Table 4:** Nomenclature of larval thoracic muscles.

Muscles	Origin	Insertion
I dam-h	antero-median area of prothoracic dorsal region	head
I dal-h	antero-lateral area of prothoracic dorsal region	head
I dml-h	meso-lateral area of prothoracic dorsal region	head
I dpl-h	postero-lateral area of prothoracic dorsal region	head
I dpm-h	postero-median area of prothoracic dorsal region	head
I d-d	prothoracic dorsal region	prothoracic dorsal region
I l-h	prothoracic lateral region	head
I l-l	prothoracic lateral region	prothoracic lateral region
I l-leg	prothoracic lateral region	basal rim of proleg
I v-h	prothoracic ventral region	head
I leg-h	basal rim of proleg	head
I leg-v	basal rim of proleg	prothoracic ventral region
I leg-leg	basal rim of proleg	basal rim of proleg
II d-d	mesothoracic dorsal region	mesothoracic dorsal region
II d-l	mesothoracic dorsal region	mesothoracic lateral region
II l-l	mesothoracic lateral region	mesothoracic lateral region
II l-v	mesothoracic lateral region	mesothoracic ventral region
II l-leg	mesothoracic lateral region	basal rim of midleg
II v-v	mesothoracic ventral region	mesothoracic ventral region
II leg-v	basal rim of midleg	mesothoracic ventral region
II leg-leg	basal rim of midleg	basal rim of midleg
III d-d	metathoracic dorsal region	metathoracic dorsal region
III d-l	metathoracic dorsal region	metathoracic lateral region
III d-v	metathoracic dorsal region	metathoracic ventral region
III l-l	metathoracic lateral region	metathoracic lateral region
III l-v	metathoracic lateral region	metathoracic ventral region
III l-leg	metathoracic lateral region	basal rim of hindleg
III v-v	metathoracic ventral region	metathoracic ventral region
III leg-v	basal rim of hindleg	metathoracic ventral region
III leg-leg	basal rim of hindleg	basal rim of hindleg
T Iv-IIl	prothoracic ventral region	mesothoracic lateral region
T Iv-IIv	prothoracic ventral region	mesothoracic ventral region
T IIl-h	mesothoracic lateral region	head
T IId-IIId	mesothoracic dorsal region	metathoracic dorsal region
T IId-IIIv	mesothoracic dorsal region	metathoracic ventral region
T IIl-IIIv	mesothoracic lateral region	metathoracic ventral region
T IIv-h	mesothoracic ventral region	head
T IIv-IIIl	mesothoracic ventral region	mesothoracic lateral region
T IIv-IIIv	mesothoracic ventral region	metathoracic ventral region
T IIId-At	metathoracic dorsal region	abdomen
T IIIl-h	metathoracic lateral region	head
T IIIv-h	metathoracic ventral region	head

**Table 5:** The thoracic muscles in each larval developmental stage. Present is denoted with “+” and green color, and absent is denoted with “−” and orange color.

Muscles	1st Instar	2nd Instar	3rd Instar	4th Instar	Prepupa
Prothorax					
I dam-h 1	+	+	+	−	−
I dam-h 2	−	+	+	+	−
I dal-h 1	+	+	+	+	+
I dal-h 2	+	+	+	+	−
I dal-h 3	−	+	+	+	+
I dal-h 4	−	+	+	+	+
I dml-h 1	+	+	+	+	−
I dml-h 2	+	+	+	−	−
I dml-h 3	−	+	+	−	−
I dml-h 4	−	+	+	−	−
I dml-h 5	−	+	−	+	−
I dpl-h 1	+	+	+	+	+
I dpl-h 2	+	+	+	+	+
I dpl-h 3	+	+	+	+	+
I dpl-h 4	−	+	+	+	+
I dpl-h 5	−	+	+	+	+
I dpl-h 6	−	+	−	+	+
I dpm-h 1	+	+	+	+	+
I d-d 1	+	−	−	−	−
I l-h 1	+	+	+	+	+
I l-h 2	+	+	+	−	+
I l-h 3	+	+	+	+	−
I l-h 4	−	+	−	−	−
I l-l 1	−	+	+	−	−
I l-l 2	−	+	+	+	+
I l-l 3	−	+	+	−	+
I l-leg 1	+	+	+	−	−
I v-h 1	−	+	+	+	−
I v-h 2	−	−	+	+	−
I leg-h 1	+	+	+	+	−
I leg-h 2	+	+	+	+	−
I leg-v 1	−	+	+	+	+
I leg-v 2	−	−	−	+	−
I leg-v 3	−	−	−	+	−
I leg-v 4	−	−	−	+	−
I leg-leg 1	+	+	+	+	−
Mesothorax					
II d-d 1	−	+	−	+	−
II d-d 2	−	+	+	+	−
II d-d 3	−	+	+	+	+
II d-l 1	−	+	−	−	−
II l-l 1	−	+	+	+	−
II l-l 2	−	+	+	−	−
II l-l 3	−	+	+	+	−
II l-l 4	−	−	+	−	−
II l-l 5	−	−	+	−	−
II l-l 6	−	−	+	+	−
II l-v 1	−	+	+	+	+
II l-v 2	−	+	−	−	−
II l-v 3	−	−	+	+	+
II l-leg 1	−	+	+	−	+
II l-leg 2	−	−	−	−	+
II l-leg 3	−	−	−	−	+
II v-v 1	−	+	+	+	+
II v-v 2	−	+	+	−	+
II v-v 3	−	−	−	+	−
II leg-v 1	−	−	−	+	−
II leg-leg 1	−	+	+	+	−
II leg-leg 2	−	−	+	+	−
II leg-leg 3	−	−	−	+	−
Metathorax					
III d-d 1	−	+	+	+	−
III d-d 2	−	+	−	+	−
III d-d 3	−	+	+	+	+
III d-d 4	−	+	+	+	+
III d-d 5	−	+	+	+	+
III d-l 1	−	+	+	+	+
III d-l 2	−	−	+	+	−
III d-v 1	−	−	−	−	+
III d-v 2	−	−	−	−	+
III d-v 3	−	−	−	−	+
III l-l 1	−	+	+	+	−
III l-l 2	−	−	+	−	+
III l-l 3	−	−	−	−	+
III l-v 1	−	−	−	−	+
III l-leg 1	−	+	+	+	+
III l-leg 2	−	+	+	+	+
III l-leg 3	−	−	+	+	+
III l-leg 4	−	−	−	−	+
III v-v 1	−	−	+	+	−
III v-v 2	−	−	+	+	+
III v-leg 1	−	+	+	+	−
III v-leg 2	−	+	+	+	−
III leg-leg 1	−	+	+	+	−
III leg-leg 2	−	−	+	+	−
Intersegmental muscles					
T Iv-IIl 1	−	−	+	−	−
T Iv-IIv 1	−	+	−	−	−
T Iv-IIv 2	−	+	−	−	−
T IId-IIId 1	−	+	−	−	−
T IId-IIIl 1	−	+	−	−	−
T IId-IIIv 1	−	+	−	−	−
T IIl-h 1	+	+	+	+	+
T IIl-h 2	+	−	+	+	−
T IIl-IIIv 1	−	+	−	−	−
T IIv-h 1	+	+	+	+	+
T IIv-IIIl 1	−	−	+	−	−
T IIv-IIIv 1	−	+	+	−	−
T IIId-At 1	−	+	−	−	−
T IIIl-h 1	−	+	+	+	+
T IIIv-h 1	−	+	+	+	+

**Table 6:** Thoracic length, width and height (µm) of the pupae and adult. “PI”, “PM” and “PL”, respectively, represent the initial, middle and late pupal stages. “A” represents the adult.

	PI	PM	PL	A
Length	1046.37	1333.79	1280.20	1390.40
Width	1102.97	1230.87	1289.23	1334.74
Height	962.00	1326.50	1264.24	1324.20

**Table 7:** The thoracic muscles in each pupal developmental stage. Present is denoted with “+” and green color, and absent is denoted with “−” and orange color.

Muscles	Initial Pupa	Middle Pupa	Late Pupa	Adult
Prothorax				
Idlm1	−	+	+	+
Idlm2	−	+	+	+
Idlm3	−	−	+	+
Idlm5	−	+	+	+
Idlm6	−	−	+	−
Idvm2	−	−	+	+
Idvm6	−	−	+	+
Idvm7	−	+	+	+
Idvm8	−	−	+	+
Idvm9	−	−	−	+
Idvm10	−	−	−	+
Idvm18	−	+	+	+
Itpm3	−	+	+	+
Itpm4	−	−	−	+
Itpm5	−	+	+	+
Itpm6	−	+	+	+
Ipcm6	−	+	+	+
Ivlm1	−	−	+	+
Ivlm3	−	−	+	+
Ivlm7	−	+	+	+
Iscm4	−	−	+	+
Mesothorax				
IIdlm1	−	+	+	+
IIdlm2	−	+	+	+
IIdvm1	−	+	+	+
IIdvm2	−	−	+	+
IItpm1	−	−	+	+
IItpm4	−	−	+	+
IIspm2	−	−	+	+
IIspm7	−	−	+	+
IIpcm4	−	+	+	+
IIvlm3	−	+	+	+
IIscm1	−	−	+	+
IIscm2	−	+	+	+
IIscm6	−	+	+	+
Metathorax				
IIIdlm1	−	+	+	+
IIIdlm2	−	+	+	+
IIIdvm1	+	+	+	+
IIIdvm2	+	+	+	+
IIIdvm4	+	+	+	+
IIIdvm5	−	+	+	+
IIIdvm8	−	+	+	+
IIItpm1	−	+	+	+
IIItpm3	−	+	+	+
IIItpm7	−	+	+	+
IIItpm8	−	−	−	+
IIItpm9	−	+	+	+
IIItpm10	−	−	−	+
IIIspm1	+	+	+	+
IIIpcm3	+	+	+	+
IIIpcm4	+	+	+	+
IIIscm1	+	+	+	+
IIIscm2	−	−	−	+
IIIscm3	−	+	+	+
IIIscm4	−	+	+	+
IIIscm6	−	+	+	+

**Table 8:** Thoracic muscles that appear only in the prepupa homologously compared with those in the initial pupa.

Muscles of Prepupa		Muscles of Initial Pupa	
III d-v 1	O (=origin): antero-lateral area of metathoracic dorsal region I (=insertion): antero-lateral area of metathoracic ventral region	IIIdvm1	O: antero-lateral area of metanotum I: antero-median area of metasternum
III d-v 2	O: antero-lateral area of metathoracic dorsal region I: antero-lateral area of metathoracic ventral region	IIIdvm2	O: latero-median area of metanotum I: latero-median area of metasternum
III d-v 3	O: postero-lateral area of metathoracic dorsal region I: postero-lateral area of metathoracic ventral region	IIIdvm4	O: antero-median area of metapleuron I: postero-lateral area of metasternum
III l-v 1	O: antero-dorsal area of metathoracic lateral region I: antero-lateral area of metathoracic ventral region	IIIspm1	O: antero-median area of metapleuron I: latero-median area of metasternum
III l-l 3	O: antero-dorsal area of metathoracic lateral region I: antero-median area of metathoracic lateral region	IIIpcm3	O: antero-median area of metapleuron I: latero-ventral margin of metacoxal rim
III l-leg 4	O: postero-ventral area of metathoracic lateral region I: antero-lateral margin of basal rim of hindleg	IIIpcm4	O: postero-median area of metapleuron I: dorso-lateral margin of metacoxal rim

## Data Availability

The original contributions presented in this study are included in the article/Supplementary Materials. Further inquiries can be directed to the corresponding authors.

## Supplementary Materials

The following supporting information can be downloaded at https://www.mdpi.com/article/10.3390/insects16030324/s1, S1: Cranial width; S2: Larval muscle description; S3: Larval muscle length and width; S4: Pupal muscle description; S5: Pupal muscle length and width.
