# Supplementary material for: Transformation of Internal Thoracic Structures of Callobruchus maculatus (Coleoptera: Bruchidae) from Larva to Adult
Source: Insects. 2025 Mar 19;16(3):324. doi: 10.3390/insects16030324 (PMC11943184; doi:10.3390/insects16030324)
Supplement: Supplementary file 1 [file insects-16-00324-s001.zip › S1 Cranial width.pdf]

Supplementary Material S1: Larval cranial width (μm)

| First instar | Second instar | Third instar | Fourth instar |
|---|---|---|---|
| 74.05 | 134.88 | 193.91 | 253.45 |
| 67.09 | 132.29 | 184.93 | 264.06 |
| 64.71 | 131.92 | 184.85 | 244.64 |
| 63.52 | 127.69 | 184.34 | 213.56 |
| 62.95 | 126.64 | 183.45 | 213.21 |
| 62.57 | 126.62 | 180.37 | 229.14 |
| 62.20 | 125.12 | 180.10 | 241.37 |
| 61.32 | 125.04 | 178.31 | 248.59 |
| 61.28 | 124.64 | 177.14 | 225.89 |
| 61.26 | 123.00 | 174.00 | 240.39 |
| 61.01 | 122.13 | 172.99 | 228.99 |
| 60.93 | 122.01 | 172.65 | 237.30 |
| 60.93 | 121.96 | 172.36 | 228.99 |
| 60.93 | 121.60 | 172.13 | 237.30 |
| 60.60 | 121.54 | 170.90 | 226.94 |
| 60.59 | 121.47 | 169.50 | 238.27 |
| 60.38 | 121.04 | 167.77 | 244.46 |
| 60.00 | 120.71 | 164.64 | 230.04 |
| 59.93 | 120.43 | 160.38 | 221.77 |
| 59.90 | 120.37 | 160.34 | 238.28 |
| 59.23 | 119.38 | 158.14 | 240.34 |
| 58.91 | 119.22 | 158.14 | 219.77 |
| 58.89 | 119.11 | 157.12 | 232.14 |
| 58.88 | 118.86 | 186.03 | 234.17 |
| 58.88 | 118.45 | 181.94 | 230.80 |
| 58.56 | 118.35 | 180.88 | 233.11 |
| 58.27 | 117.84 | 174.15 | 244.46 |
| 58.19 | 116.66 | 171.62 | 258.12 |
| 58.02 | 116.65 | 166.17 | 227.53 |
| 57.85 | 116.65 | 165.89 | 231.83 |
| 57.61 | 116.63 | 164.85 | 219.30 |
| 57.53 | 116.36 | 157.62 | 217.65 |
| 57.51 | 116.31 | 157.10 | 235.69 |

| | | | |
|---|---|---|---|
| 57.51 | 116.27 | 155.67 | 242.40 |
| 57.16 | 115.83 | 154.52 | 221.85 |
| 56.82 | 115.81 | 154.52 | 224.87 |
| 56.55 | 115.76 | 153.49 | 228.99 |
| 56.52 | 115.70 | 140.57 | 248.66 |
| 56.48 | 115.70 | | 226.29 |
| 56.14 | 115.56 | | 233.11 |
| 55.96 | 115.25 | | 236.29 |
| 55.81 | 114.55 | | 218.70 |
| 55.79 | 114.55 | | 230.03 |
| 55.45 | 114.55 | | 224.92 |
| 55.11 | 114.40 | | 242.42 |
| 54.84 | 114.32 | | 215.64 |
| 54.74 | 114.15 | | 239.31 |
| 54.44 | 113.33 | | 249.65 |
| 53.75 | 112.80 | | 232.30 |
| 53.06 | 112.45 | | 241.35 |
| 51.35 | 112.18 | | 247.62 |
| | 112.17 | | 236.27 |
| | 112.14 | | 233.15 |
| | 110.80 | | 229.49 |
| | 108.32 | | 226.93 |
| | 108.05 | | 224.86 |
| | 105.94 | | 221.23 |
| | 102.81 | | 220.00 |
| | 101.54 | | 219.74 |
| | 99.44 | | 219.72 |
| | 107.05 | | 219.03 |
| | 99.29 | | 218.83 |
| | 86.26 | | 216.64 |
| | 83.92 | | 215.70 |
| | | | 215.57 |
| | | | 214.55 |
| | | | 214.14 |
| | | | 213.68 |
| | | | 211.61 |
| | | | 211.36 |

| | | | |
|---|---|---|---|
| | | | 207.33 |
| | | | 205.27 |
| | | | 196.71 |
