# Supplementary material for: Transformation of Internal Thoracic Structures of Callobruchus maculatus (Coleoptera: Bruchidae) from Larva to Adult
Source: Insects. 2025 Mar 19;16(3):324. doi: 10.3390/insects16030324 (PMC11943184; doi:10.3390/insects16030324)
Supplement: Supplementary file 1 [file insects-16-00324-s001.zip › S2 Larval muscle description.pdf]

Supplementary Material S2: Larva musculature

Prothoracic muscles

I dam-h 1

First instar: O (= origin): antero-median area of prothoracic dorsal region. I (= insertion): postero-median area of vertex. Long triangle, narrowing towards head, slightly bent dorsad.

Second instar: Bent dorsad.

Third instar: Straight.

Fourth instar: Absent.

Prepupa: Absent

Developmental changes: The muscle is more bent in the second instar and straight in the third instar.

I dam-h 2

First instar: Absent.

Second instar: O: antero-median area of prothoracic dorsal region. I: postero-median area of vertex. Broad medially and narrowing towards both ends, slightly bent antero-dorsad.

Third instar: Parallelogram, bent laterad.

Fourth instar: Long triangle, narrowing towards head, slightly bent dorsad.

Prepupa: Absent.

Developmental changes: The original end becomes broader in the third instar. The insertional end becomes broader in the third instar and narrower in the fourth instar.

I dal-h 1

First instar: O: antero-lateral area of prothoracic dorsal region. I: postero-median area of vertex. Long triangle, narrowing towards head, slightly bent dorsad.

Second instar: Bent dorsad.

Third instar: Approximate parallelogram, original end narrower than insertional end, straight.

Fourth instar: Parallelogram, curved.

Prepupa: Approximate long triangle, narrowing towards prothoracic dorsal region, curved.

Developmental changes: The original end becomes narrower in the prepupa. The insertional end becomes broader in the third instar. The muscle is more bent in the second instar, straight in the third instar and curved in the fourth instar.

I dal-h 2

First instar: O: antero-lateral area of prothoracic dorsal region. I: postero-median area of vertex. Approximate parallelogram, original end narrower than insertional end, curved.

Second instar: Almost the same as the last stage.
Third instar: Straight.
Fourth instar: Long triangle, narrowing towards prothoracic dorsal region, curved.
Prepupa: Absent.
Developmental changes: The original end becomes narrower in the fourth instar. The muscle is straight in the third instar and curved in the other stages.

I dal-h 3
First instar: Absent.
Second instar: O: antero-lateral area of prothoracic dorsal region. I: postero-median area of vertex. Broad medially and narrowing towards both ends, straight.
Third instar: Long triangle, narrowing towards prothoracic dorsal region, straight.
Fourth instar: Broad medially and narrowing towards both ends, slightly bent antero-proximad.
Prepupa: O: antero-lateral area of prothoracic dorsal region. I: postero-lateral area of head. Long triangle, narrowing towards head, bent laterad.
Developmental changes: The original end becomes broader in the prepupa. The insertional end becomes broader in the third instar and narrower in the fourth instar. The muscle is straight in the second and third instars, slightly bent antero-proximad in the fourth instar and bent laterad in the prepupa.

I dal-h 4
First instar: Absent.
Second instar: O: antero-lateral area of prothoracic dorsal region. I: postero-median area of vertex. Approximate long triangle, narrowing towards head, slightly bent dorsad.
Third instar: Long triangle, narrowing towards prothoracic dorsal region, straight.
Fourth instar: Broad medially and narrowing towards both ends, slightly bent dorsad.
Prepupa: Broad medially and narrowing towards both ends, slightly bent posterad.
Developmental changes: The original end becomes narrower in the third instar. The insertional end becomes broader in the third instar and narrower in the fourth instar. The muscle is slightly bent dorsad in the second instar and the fourth instar, straight in the third instar and slight bent posterad in the prepupa.

I dml-h 1
First instar: O: meso-lateral area of prothoracic dorsal region. I: extends towards head. Flat triangle, narrowing towards head, straight.
Second instar: O: meso-lateral area of prothoracic dorsal region. I: postero-lateral area of vertex. Broad medially and narrowing towards both ends, slightly bent postero-proximad.
Third instar: Parallelogram, straight.

Fourth instar: Slightly bent postero-laterad.

Prepupa: Absent.

Developmental changes: The muscle extends postero-ventrad until connects with the head in the second instar. The original end becomes narrower in the second instar. The insertional end becomes broader in the fourth instar. The muscle is straight in the first and third instars, slightly bent postero-proximad in the second instar and slightly bent postero-laterad in the fourth instar.

I dml-h 2

First instar: O: antero-lateral area of prothoracic dorsal region. I: postero-median area of vertex. Long triangle, narrowing towards head, slightly bent dorsad.

Second instar: Bent dorso-proximad.

Third instar: Curved.

Fourth instar: Absent.

Prepupa: Absent.

Developmental changes: The muscle is slightly bent dorsad in the first instar, bent dorso-proximad in the second instar and curved in the third instar.

I dml-h 3

First instar: Absent.

Second instar: O: meso-lateral area of prothoracic dorsal region. I: postero-lateral area of head. Broad medially and narrowing towards both ends, straight.

Third instar: Slightly bent postero-proximad.

Fourth instar: Absent.

Prepupa: Absent.

Developmental changes: The muscle is straight in the second instar and slightly bent postero-proximad in the third instar.

I dml-h 4

First instar: Absent.

Second instar: O: meso-lateral area of prothoracic dorsal region. I: postero-lateral area of head. Long triangle, narrowing towards head, straight.

Third instar: O: meso-lateral area of prothoracic dorsal region. I: meso-lateral area of head. Approximate long triangle, narrowing towards head, slightly bent postero-proximad.

Fourth instar: Absent.

Prepupa: Absent.

Developmental changes: The insertional end moves anterad in the third instar. The muscle is straight in the second instar and slightly bent postero-proximad in the third instar.

I dml-h 5

First instar: Absent.

Second instar: O: meso-lateral area of prothoracic dorsal region. I: postero-lateral area of head. Parallelogram, slightly bent proximad.

Third instar: Absent.

Fourth instar O: meso-lateral area of prothoracic dorsal region. I: lateral area of head. Approximate long triangle, narrowing towards head, straight.

Prepupa Absent.

Developmental changes: The insertional end moves anterad in the fourth instar. The insertional end becomes narrower. The muscle is slightly bent proximad in the second instar and straight in the fourth instar.

I dpl-h 1

First instar: O: meso-lateral area of prothoracic dorsal region. I: postero-lateral area of head. Broad medially and narrowing towards both ends, straight.

Second instar: O: postero-lateral area of prothoracic dorsal region. I: postero-lateral area of head. Long triangle, narrowing towards head, straight.

Third instar: Parallelogram, slightly bent postero-proximad.

Fourth instar: O: postero-lateral area of prothoracic dorsal region. I: lateral area of head. Long triangle, narrowing towards head, slightly bent postero-proximad.

Prepupa: O: postero-lateral area of prothoracic dorsal region. I: postero-lateral area of head. Broad medially and narrowing towards both ends, curved.

Developmental changes: The original end moves posterad in the second instar. The insertional end moves anterad in the fourth instar and posterad in the prepupa. The original end becomes broader in the second instar and narrower in the prepupa. The insertional end becomes broader in the third instar and narrower in the fourth instar. The muscle is straight in the first and second instars, bent postero-proximad in the third instar and fourth instar and cured in the prepupa.

I dpl-h 2

First instar: O: postero-lateral area of prothoracic dorsal region. I: postero-lateral area of vertex. Parallelogram, bent ventro-laterad.

Second instar: Long triangle, narrowing towards prothoracic dorsal region, straight.

Third instar: Approximate parallelogram, original end narrower than insertional end, straight.

Fourth instar: Isosceles long triangle, narrowing towards head.

Prepupa: Broad medially and narrowing towards both ends, curved.

Developmental changes: The original end becomes narrower in the second instar and prepupa and broader in the third instar. The insertional end becomes narrower in the fourth instar. The muscle is bent ventro-laterad in the first instar, straight in the second, third instars

and the fourth instar and curved prepupa

I dpl-h 3

First instar: O: postero-lateral area of prothoracic dorsal region. I: postero-lateral area of vertex. Isosceles long triangle, narrowing towards head, straight.

Second instar: Long triangle, narrowing towards prothoracic dorsal region, bent ventro-posterad.

Third instar: Approximate parallelogram, original end narrower than insertional end, slightly bent ventrad.

Fourth instar: Isosceles long triangle, narrowing towards head, straight.

Prepupa: Almost the same as the last stage.

Developmental changes: The original end becomes narrower in the second instar and broader in the third instar. The insertional end becomes broader in the second instar. The muscle is straight in the first and fourth instars, bent ventro-posterad in the second instar and slightly bent ventrad in the third instar.

I dpl-h 4

First instar: Absent.

Second instar: O: postero-lateral area of prothoracic dorsal region. I: postero-lateral area of vertex. Trapezoid, original end broader than insertional end, straight.

Third instar: Long triangle, narrowing towards head, slightly bent ventrad.

Fourth instar: O: postero-lateral area of prothoracic dorsal region. I: meso-ventral area of lateral side of head. Approximate parallelogram, original end broader than insertional end, straight.

Prepupa: O: postero-lateral area of prothoracic dorsal region. I: postero-lateral area of vertex. Bent dorsad.

Developmental changes: The insertional end moves antero-ventrad in the fourth instar C-type and postero-dorsad in the prepupa. The insertional end becomes narrower in the third instar and broader in the fourth instar. The muscle is straight in the second instar and the fourth instar, slightly bent ventrad in the third instar and bent dorsad in the prepupa.

I dpl-h 5

First instar: Absent.

Second instar: O: postero-lateral area of prothoracic dorsal region. I: postero-lateral area of head. Broad medially and narrowing towards both ends, straight.

Third instar: Long triangle, narrowing towards head, slightly bent dorso-proximad.

Fourth instar: Almost the same as the last stage.

Prepupa: Approximate parallelogram, original and insertional ends equal, curved.

Developmental changes: The original end becomes broader in the third instar. The

insertional end becomes broader in the prepupa. The muscle is straight in the second instar, slightly bent dorso-proximad in the third instar and in the fourth instar and curved in the prepupa.

I dpl-h 6

First instar: Absent.

Second instar: O: postero-lateral area of prothoracic dorsal region. I: meso-ventral area of lateral area of head. Broad medially and narrowing towards both ends, slightly bent antero-laterad.

Third instar: Absent.

Fourth instar: Approximate parallelogram, original end broader than insertional end, straight.

Prepupa: Approximate parallelogram, original end narrower than insertional end, curved.

Developmental changes: Both original and insertional ends become broader in the fourth instar. The muscle is slightly bent antero-laterad in the second instar, straight in the fourth instar and curved in the prepupa.

I dpm-h 1

First instar: O: postero-median area of prothoracic dorsal region. I: extends towards head. Broad medially and narrowing towards both ends, straight.

Second instar: O: postero-median area of prothoracic dorsal region. I: postero-lateral area of vertex. Long triangle, narrowing towards prothoracic dorsal region, bent antero-proximad.

Third instar: Approximate parallelogram, both original and insertional ends equal, slightly bent antero-proximad.

Fourth instar: Long triangle, narrowing towards head, straight.

Prepupa: Approximate long triangle, narrowing towards prothoracic dorsal region.

Developmental changes: The muscle extends ventro-posterad until connects with the head in the second instar. The original end becomes broader in the third instar and narrower in the prepupa. The insertional end becomes broader in the second instar and broader in the third instar. The muscle is straight in the first and fourth instars and bent antero-proximad in the second and third instars.

I d-d 1

First instar: O: meso-lateral area of prothoracic dorsal region. I: meso-lateral area of prothoracic dorsal region. Long triangle, narrowing towards antero-ventrad, straight.

Second instar: Absent.

Third instar: Absent.

Fourth instar: Absent.

Prepupa: Absent.

Developmental changes: Not application.

I I-h 1

First instar: O: postero-median area of prothoracic lateral region. I: extends towards head. Trapezoid, original end narrower than insertional end, straight.

Second instar: O: postero-median area of prothoracic lateral region. I: meso-lateral area of head. Approximate long triangle, narrowing towards prothoracic lateral region.

Third instar: Long triangle, narrowing towards head, slightly bent laterad.

Fourth instar O: antero-median area of prothoracic lateral region. I: meso-lateral area of head. Slightly bent antero-proximad.

Prepupa: O: postero-ventral area of prothoracic lateral region. I: potero-lateral area of head. Curved.

Developmental changes: The muscle extends meso-proximad until connects with the head in the second instar. The original end moves anterad in the fourth instar and postero-ventrad in the prepupa. The original end becomes narrower in the second instar and broader in the third instar. The insertional end becomes narrower in the third instar. The muscle is straight in the first and second instars, slightly bent laterad in the third instar, slightly bent antero-proximad in the fourth instar and curved in the prepupa.

I I-h 2

First instar: O: postero-dorsal area of prothoracic lateral region. I: postero-lateral area of head. Long triangle, narrowing towards head, straight.

Second instar: O: antero-dorsal area of prothoracic lateral region. I: postero-lateral area of head. Parallelogram, bent postero-ventrad.

Third instar: O: postero-dorsal area of prothoracic lateral region. I: postero-lateral area of head. Long triangle, narrowing towards prothoracic lateral region, straight.

Fourth instar: Absent.

Prepupa: Long triangle, narrowing towards head, straight.

Developmental changes: The original end moves anterad in the second instar and posterad in the third instar. The original end becomes narrower in the third instar and broader in the prepupa. The insertional end becomes broader in the second instar and narrower in the prepupa.

I I-h 3

First instar: O: postero-dorsal area of prothoracic lateral region. I: postero-lateral area of head. Long triangle, narrowing towards head, straight.

Second instar: O: meso-ventral area of prothoracic lateral region. I: postero-ventral area of lateral area of head.

Third instar: Long triangle, narrowing towards prothoracic lateral region.

Fourth instar: Long triangle, narrowing towards head.

Prepupa: Absent.

Developmental changes: The original end moves antero-ventrad in the second instar. The insertional end moves ventrad. The original end becomes narrower in the third instar and broader in the fourth instar. The insertional end becomes broader in the third instar.

I I-h 4

First instar: Absent.

Second instar: O: antero-median area of prothoracic lateral region. I: postero-lateral area of vertex. Approximate parallelogram, original end narrower than insertional end, straight.

Third instar: Absent.

Fourth instar: Absent.

Prepupa: Absent.

Developmental changes: Not applicable.

I I-l 1

First instar: Absent.

Second instar: O: antero-dorsal area of prothoracic lateral region. I: meso-dorsal area of prothoracic lateral region. Broad medially and narrowing towards both ends, straight.

Third instar: Slightly bent postero-proximad.

Fourth instar: Absent.

Prepupa: Absent.

Developmental changes: The muscle is straight in the second instar and slightly bent postero-proximad in the third instar.

I I-l 2

First instar: Absent.

Second instar: O: antero-dorsal area of prothoracic lateral region. I: postero-dorsal area of prothoracic lateral region. Parallelogram, straight.

Third instar: O: antero-median area of prothoracic lateral region. I: postero-dorsal area of prothoracic lateral region. Approximate parallelogram, both original and insertional ends equal, bent postero-proximad.

Fourth instar: Approximate parallelogram, both original and insertional ends equal, straight.

Prepupa: O: antero-ventral area of prothoracic lateral region. I: meso-dorsal area of prothoracic lateral region. Approximate long triangle, narrowing towards antero-dorsad.

Developmental changes: The original end moves ventrad from the second instar to the fourth instar. The insertional end moves anterad in the prepupa. The original end becomes narrower in the prepupa. The muscle is straight in the second and fourth instars and bent postero-proximad in the third instar.

I l-l 3

First instar: Absent.

Second instar: O: antero-ventral area of prothoracic lateral region. I: meso-dorsal area of prothoracic lateral region. Broad medially and narrowing towards both ends, straight.

Third instar: O: meso-ventral area of prothoracic lateral region. I: meso-dorsal area of prothoracic lateral region.

Fourth instar: Absent.

Prepupa: O: postero-ventral area of prothoracic lateral region. I: meso-dorsal area of prothoracic lateral region.

Developmental changes: The original end moves posterad from the second instar to the fourth instar.

I l-leg 1

First instar: O: meso-ventral area of prothoracic lateral region. I: meso-lateral area of prothoracic ventral region. Approximate parallelogram, both original and insertional ends equal, slightly bent ventro-proximad.

Second instar: O: meso-dorsal area of prothoracic lateral region. I: antero-lateral margin of basal rim of proleg. Straight.

Third instar: O: meso-ventral area of prothoracic lateral region. I: antero-lateral margin of basal rim of proleg.

Fourth instar: Absent.

Prepupa: Absent.

Developmental changes: The original end moves dorsad in the second instar and ventrad in the third instar. The muscle is slightly bent ventro-proximad in the first instar and straight in the second and third instars.

I v-h 1

First instar: Absent.

Second instar: O: antero-lateral area of prothoracic ventral region. I: postero-ventral area of lateral area of head. Approximate long triangle, narrowing towards head, straight.

Third instar: Almost the same as the last stage.

Fourth instar: O: antero-lateral area of prothoracic ventral region. I: meso-ventral area of lateral area of head. Broadly medially and narrowing towards both ends, bent postero-laterad.

Prepupa: Absent.

Developmental changes: The insertional end moves anterad in the fourth instar. The original end becomes narrower in the fourth instar. The muscle is straight in the second and third instars and bent postero-laterad.

I v-h 2

First instar: Absent.

Second instar: Absent.

Third instar: O: antero-lateral area of prothoracic ventral region. I: meso-ventral area of lateral area of head. Broad medially and narrowing towards both ends, straight.

Fourth instar: Long triangle, narrowing towards head.

Prepupa: Absent.

Developmental changes: The insertional end becomes narrower in the fourth instar.

I leg-h 1

First instar: O: meso-lateral area of prothoracic ventral region. I: meso-ventral area of lateral area of head. Long triangle, narrowing towards head, straight.

Second instar: O: antero-lateral margin of basal rim of proleg. I: meso-ventral area of lateral area of head. Slightly bent postero-laterad.

Third instar: Parallelogram, straight.

Fourth instar: Approximate parallelogram, original end broader than insertional end.

Prepupa: Absent.

Developmental changes: The insertional end becomes narrower in the fourth instar. The muscle is straight in the first, second and fourth instar and slightly bent postero-laterad in the second instar.

I leg-h 2

First instar: O: postero-lateral area of prothoracic ventral region. I: postero-lateral area of head. Approximate parallelogram, both original and insertional ends equal, slightly bent antero-ventrad.

Second instar: O: postero-lateral margin of basal rim of proleg. I: postero-lateral area of head. Long triangle, narrowing towards head.

Third instar: Approximate parallelogram, original end narrower than insertional end.

Fourth instar: Parallelogram, straight.

Prepupa: Absent.

Developmental changes: The insertional end becomes narrower in the second instar and broader in the third instar. The muscles is slightly bent antero-ventrad in the first to third instars and straight in the fourth instar.

I leg-v 1

First instar: Absent.

Second instar: O: postero-proximal margin of basal rim of proleg. I: antero-median area of prothoracic ventral region. Long triangle, narrowing towards proleg, straight.

Third instar: Approximate parallelogram, original end broader than insertional end.

Fourth instar: Almost the same as the last stage.

Prepupa: Parallelogram, slightly bent dorsad.
Developmental changes: The insertional end becomes broader in the third instar. The muscle is straight from the second instar to the fourth instar and slightly bent dorsad in the prepupa.

I leg-v 2
First instar: Absent.
Second instar: Absent.
Third instar: Absent.
Fourth instar: O: posterior margin of basal rim the proleg. I: antero-median area of prothoracic ventral region. Long triangle, narrowing towards proleg, straight.
Prepupa: Absent.
Developmental changes: Not applicable.

I leg-v 3
First instar: Absent.
Second instar: Absent.
Third instar: Absent.
Fourth instar: O: postero-proximal margin of basal rim of proleg. I: antero-lateral area of prothoracic ventral region. Parallelogram, slightly bent dorso-posterad.
Prepupa: Absent.
Developmental changes: Not applicable.

I leg-v 4
First instar: Absent.
Second instar: Absent.
Third instar: Absent.
Fourth instar O: postero-proximal margin of basal rim of proleg. I: antero-lateral area of prothoracic ventral region. Long triangle, narrowing towards prothoracic ventral region, straight.
Prepupa: Absent.
Developmental changes: Not applicable.

I leg-leg 1
First instar: O: meso-lateral area of prothoracic ventral region. I: antero-lateral area of prothoracic ventral region. Long triangle, narrowing towards anterad, straight.
Second instar: O: postero-proximal margin of basal rim of proleg. I: antero-proximal margin of basal rim of proleg. Slightly bent dorsad.
Third instar: Approximate parallelogram, original end broader than insertional end, straight.
Fourth instar: O: postero-proximal margin of basal rim of proleg. I: antero-lateral margin of

basal rim of proleg.

Prepupa: Absent.

Developmental changes: The insertional end is broader in the third instar. The muscle is straight in the first, third and fourth instars and slightly bent dorsad in the second instar.

Mesothoracic muscles

II d-d 1

First instar: Absent.

Second instar: O: antero-median area of mesothoracic dorsal region. I: postero-median area of mesothoracic dorsal region. Parallelogram, slightly bent postero-ventrad.

Third instar: Absent.

Fourth instar: Approximate parallelogram, original end broader than insertional end, straight.

Prepupa: Absent.

Developmental changes: The muscle is slightly bent postero-ventrad and straight in the fourth instar.

II d-d 2

First instar: Absent.

Second instar: O: antero-lateral area of mesothoracic dorsal region. I: postero-median area of mesothoracic dorsal region. Long triangle, narrowing towards postero-proximad, straight.

Third instar: O: antero-lateral area of mesothoracic dorsal region. I: postero-lateral area of mesothoracic dorsal region. Long triangle, narrowing antero-laterad.

Fourth instar: Approximate parallelogram, original end narrower than insertional end.

Prepupa: Approximate parallelogram, both original and insertional ends equal, curved.

Developmental changes: The original end becomes narrower in the third instar and broader in the fourth instar. The insertional end becomes broader in the third instar. The muscle is straight from the second instar to the fourth instar and curved in the prepupa.

II d-d 3

First instar: Absent.

Second instar: O: antero-lateral area of mesothoracic dorsal region. I: postero-lateral area of mesothoracic dorsal region. Long triangle, narrowing antero-laterad, straight.

Third instar: Almost the same as the last stage.

Fourth instar: Approximate parallelogram, original end narrower than insertional end.

Prepupa: Approximate parallelogram, both original and insertional ends equal, curved.

Developmental changes: The insertional end becomes broader in the fourth instar. The muscle is straight from the second instar to the fourth instar and curved in the prepupa.

II d-l 1

First instar: Absent.

Second instar: O: postero-lateral area of mesothoracic dorsal region. I: antero-dorsal area of mesothoracic lateral region. Parallelogram, straight.

Third instar: Absent.

Fourth instar: Absent.

Prepupa: Absent.

Developmental changes: Not applicable.

II l-l 1

First instar: Absent.

Second instar: O: antero-dorsal area of mesothoracic lateral region. I: postero-median area of mesothoracic lateral region. Parallelogram, straight.

Third instar: Approximate parallelogram, original end broader than insertional end, slightly bent laterad.

Fourth instar: Approximate parallelogram, original end narrower than insertional end, slightly bent proximad.

Prepupa: Absent.

Developmental changes: The muscle is straight in the second instar, slightly bent laterad in the third instar and slightly bent proximad in the fourth instar.

II l-l 2

First instar: Absent.

Second instar: O: antero-dorsal area of mesothoracic lateral region. I: postero-dorsal area of mesothoracic lateral region. Long triangle, narrowing anterad, straight.

Third instar: Approximate parallelogram, original end narrower than insertional end, bent proximad.

Fourth instar: Absent.

Prepupa: Absent.

Developmental changes: The original end becomes broader in the third instar. The muscle is straight in the second instar and bent proximad in the third instar.

II l-l 3

First instar: Absent.

Second instar: O: antero-dorsal area of mesothoracic lateral region. I: postero-dorsal area of mesothoracic lateral region. Approximate parallelogram, original end broader than insertional end, straight.

Third instar: Almost the same as the last stage.

Fourth instar: Slightly bent proximad.

Prepupa: Absent.

Developmental changes: The muscle is straight in the second and third instars and slightly bent proximad in the fourth instar.

II l-l 4

First instar: Absent.

Second instar: Absent.

Third instar: O: antero-dorsal area of mesothoracic lateral region. I: postero-dorsal area of mesothoracic lateral region. Approximate parallelogram, original end broader than insertional end, slightly bent laterad.

Fourth instar: Absent.

Prepupa: Absent.

Developmental changes: Not applicable.

II l-l 5

First instar: Absent.

Second instar: Absent.

Third instar: O: antero-dorsal area of mesothoracic lateral region. I: postero-median area of mesothoracic lateral region. Approximate parallelogram, original end narrower than insertional end, straight.

Fourth instar: Absent.

Prepupa: Absent.

Developmental changes: Not applicable.

II l-l 6

First instar: Absent.

Second instar: Absent.

Third instar: O: antero-ventral area of mesothoracic lateral region. I: postero-dorsal area of mesothoracic region. Long triangle, narrowing antero-ventrad, bent proximad.

Fourth instar: Approximate parallelogram, original end broader than insertional end, slightly bent proximad.

Prepupa: Absent.

Developmental changes: The insertional end becomes broader in the fourth instar.

II l-v 1

First instar: Absent.

Second instar: O: postero-median area of mesothoracic lateral region. I: antero-lateral area of mesothoracic ventral region. Long triangle, narrowing towards mesothoracic lateral region, slightly bent postero-laterad.

Third instar: Slightly bent antero-proximad.

Fourth instar: Slightly bent antero-laterad.

Prepupa: O: postero-dorsal area of mesothoracic lateral region. I: postero-lateral area of mesothoracic ventral region. Approximate parallelogram, original end broader than insertional end, curved.

Developmental changes: The original end moves dorsad in the prepupa. The insertional end moves posterad in the prepupa. The original end becomes broader in the prepupa. The muscle is slightly bent postero-laterad in the second instar, slightly bent antero-proximad in the third instar, slightly bent antero-laterad in the fourth instar and curved in the prepupa.

II l-v 2

First instar: Absent.

Second instar: O: postero-ventral area of mesothoracic lateral area. I: antero-median area of mesothoracic ventral area. Approximate parallelogram, both original and insertional ends equal, bent postero-laterad.

Third instar: Absent.

Fourth instar: Absent.

Prepupa: Absent.

Developmental changes: Not applicable.

II l-v 3

First instar: Absent.

Second instar: Absent.

Third instar: O: antero-ventral area of mesothoracic lateral region. I: postero-lateral area of mesothoracic ventral region. Parallelogram, straight.

Fourth instar: Approximate parallelogram, original end narrower than insertional end.

Prepupa: Broad medially and narrowing towards both ends.

Developmental changes: Both original and insertional ends become narrower in the prepupa.

II l-leg 1

First instar: Absent.

Second instar: O: antero-dorsal area of mesothoracic lateral region. I: antero-lateral margin of basal rim of midleg. Parallelogram, straight.

Third instar: O: meso-dorsal area of mesothoracic lateral region. I: meso-lateral margin of basal rim of midleg. Approximate parallelogram, both original and insertional ends equal.

Fourth instar: Absent.

Prepupa: O: antero-dorsal area of mesothoracic lateral region. I: postero-lateral margin of basal rim of midleg. Approximate parallelogram, both original and insertional ends equal, straight.

Developmental changes: The original end moves posterad in the third instar and anterad in the prepupa. The insertional end moves posterad from second instar to prepupa

II l-leg 2

First instar: Absent.

Second instar: Absent.

Third instar: Absent.

Fourth instar: Absent.

Prepupa: O: antero-dorsal area of mesothoracic lateral region. I: postero-lateral margin of basal rim of midleg. Approximate parallelogram, original end narrower than insertional end, slightly bent antero-ventrad.

Developmental changes: Not applicable.

II l-leg 3

First instar: Absent.

Second instar: Absent.

Third instar: Absent.

Fourth instar: Absent.

Prepupa: O: postero-dorsal area of mesothoracic lateral region. I: postero-lateral margin of basal rim of midleg. Broad medially and narrowing towards both ends, bent antero-ventrad.

Developmental changes: Not applicable.

II v-v 1

First instar: Absent.

Second instar: O: antero-lateral area of mesothoracic ventral region. I: postero-median area of mesothoracic ventral region. Broad medially and narrowing towards both ends, straight.

Third instar: Approximate parallelogram, original end narrower than insertional end.

Fourth instar: O: antero-lateral area of mesothoracic ventral region. I: postero-lateral area of mesothoracic ventral region. Slightly bent dorso-posterad.

Prepupa: O: antero-median area of mesothoracic ventral region. I: postero-lateral area of mesothoracic ventral region.

Developmental changes: Both original and insertional ends become broader in the third instar. The muscle is straight in the second and third instars and slightly bent dorso-posterad in the fourth instar.

II v-v 2

First instar: Absent.

Second instar: O: meso-lateral area of mesothoracic ventral region. I: postero-lateral area of mesothoracic ventral region. Approximate long triangle, narrowing antero-laterad, straight.

Third instar: Almost the same as the last stage.

Fourth instar: Absent.

Prepupa: O: antero-lateral area of mesothoracic ventral region. I: postero-lateral area of mesothoracic ventral region. Slightly bent proximad.

Developmental changes: The original end moves anterad in the prepupa. The muscle is straight in the second and third instars and slightly bent proximad in the prepupa.

II v-v 3

First instar: Absent.

Second instar: Absent.

Third instar: Absent.

Fourth instar: O: antero-lateral area of mesothoracic ventral region. I: postero-lateral area of mesothoracic ventral region. Parallelogram, slightly bent dorsad.

Prepupa: Absent.

Developmental changes: Not applicable.

II leg-v 1

First instar: Absent.

Second instar: Absent.

Third instar: Absent.

Fourth instar: O: posterior margin of basal rim of midleg. I: antero-median area of mesothoracic ventral region. Approximate parallelogram, original end broader than insertional end, straight.

Prepupa: Absent.

Developmental changes: Not applicable.

II leg-leg 1

First instar: Absent.

Second instar: O: antero-lateral margin of basal rim of midleg. I: postero-lateral margin of basal rim of midleg. Long triangle, narrowing antero-laterad, straight.

Third instar: O: antero-proximal margin of basal rim of midleg. I: postero-lateral margin of basal rim of midleg. Parallelogram.

Fourth instar: O: antero-lateral margin of basal rim of midleg. I: postero-lateral mrgin of basla rim of midleg. Approximate parallelogram, original end broader than insertional end, slightly bent postero-dorsad.

Prepupa: Absent.

Developmental changes: The original end moves proximad in the third instar and laterad in the fourth instar. The original end become broader in the third instar. The muscle is straight in the second and third instars and slightly bent postero-dorsad in the fourth instar.

II leg-leg 2
First instar: Absent.
Second instar: Absent.
Third instar: O: antero-lateral margin of basal rim of midleg. I: postero-proximal margin of basal rim of midleg. Approximate parallelogram, original end broader than insertional end, straight.
Fourth instar: O: antero-proximal margin of basal rim of midleg. I: postero-lateral margin of basal rim of midleg. Approximate parallelogram, original end narrower than insertional end, slightly bent dorsad.
Prepupa: Absent.
Developmental changes: The original end moves proximad in the fourth instar. The insertional end moves laterad in the fourth instar. The muscle is straight in the third instar and slightly bent dorsad in the fourth instar.

II leg-leg 3
First instar: Absent.
Second instar: Absent.
Third instar: Absent.
Fourth instar: O: antero-lateral margin of basal rim of midleg. I: postero-proximal margin of basal rim of midleg. Broad medially and narrowing towards both ends, slightly bent dorsad.
Prepupa: Absent.
Developmental changes: Not applicable.

Metathoracic muscles

III d-d 1
First instar: Absent.
Second instar: O: antero-median area of metathoracic dorsal region. I: postero-lateral area of metathoracic dorsal region. Parallelogram, straight.
Third instar: O: antero-lateral area of metathoracic dorsal region. I: postero-lateral area of metathoracic dorsal region. Approximate parallelogram, original end broader than insertional end, slightly bent ventro-proximad.
Fourth instar: Approximate parallelogram, original end narrower than insertional end, straight.
Prepupa: Absent.
Developmental changes: The original end moves laterad in the third instar. The muscle is straight in the second instar and the fourth instar, and slightly bent ventro-proximad in the third instar.

III d-d 2

First instar: Absent.

Second instar: O: antero-lateral area of metathoracic dorsal region. I: postero-lateral area of metathoracic dorsal region. Broad medially and narrowing towards both ends, straight.

Third instar: Absent.

Fourth instar: Broad anteriorly and narrow posteriorly.

Prepupa: Absent.

Developmental changes: The original end becomes narrower in the fourth instar.

III d-d 3

First instar: Absent.

Second instar: O: antero-lateral area of metathoracic dorsal region. I: postero-lateral area of metathoracic dorsal region. Approximate parallelogram, original end narrower than insertional end, slightly bent ventro-proximad.

Third instar: Almost the same as the last stage.

Fourth instar: Curved.

Prepupa: Slightly bent ventro-proximad.

Developmental changes: The muscle is slightly bent ventro-proximad in the second and third instars and the prepupa, and curved in the fourth instar.

III d-d 4

First instar: Absent.

Second instar: O: meso-lateral area of metathoracic dorsal region. I: postero-lateral area of metathoracic dorsal region. Long triangle, narrowing postero-laterad, straight.

Third instar: O: antero-lateral area of metathoracic dorsal region. I: postero-lateral area of metathoracic dorsal region. Long triangle, narrowing towards postero-laterad, bent ventro-proximad.

Fourth instar: Approximate parallelogram, original end narrower than insertional end, slightly bent ventro-proximad.

Prepupa: Approximate parallelogram, both original and insertional ends equal, straight.

Developmental changes: The original end moves anterad in the third instar. The insertional end becomes broader in the fourth instar. The muscle is straight in the second instar and the prepupa, and bent ventro-proximad in the third and fourth instars

III d-d 5

First instar: Absent.

Second instar: O: antero-lateral area of metathoracic dorsal region. I: postero-lateral area of metathoracic dorsal region. Approximate parallelogram, original end narrower than

insertional end, straight.

Third instar: Bent ventro-proximad.

Fourth instar: Narrow medially and broadening towards both ends, slightly bent ventro-proximad.

Prepupa: Parallelogram, slightly bent dorso-laterad.

Developmental changes: The muscle is straight in the second instar, bent ventro-proximad in the third and fourth instars, and slightly bent dorso-laterad in the prepupa.

III d-l 1

First instar: Absent.

Second instar: O: postero-lateral area of metathoracic dorsal region. I: antero-dorsal area of metathoracic lateral region. Broad medially and narrowing towards both ends, slightly bent postero-ventrad.

Third instar: O: postero-lateral area of metathoracic dorsal region. I: antero-median area of metathoracic lateral region. Approximate parallelogram, original end narrower than insertional end, slightly bent proximad.

Fourth instar: Approximate parallelogram, original end narrower than insertional end, slightly bent postero-ventrad.

Prepupa: O: postero-lateral area of metathoracic dorsal region. I: postero-ventral area of metathoracic lateral region. Parallelogram, bent anterad.

Developmental changes: The insertional end moves ventrad in the third instar and postero-ventrad in the prepupa. The muscle is slight bent postero-ventrad in the second instar and the fourth instar, slightly bent proximad in the third instar, and bent anterad in the prepupa.

III d-l 2

First instar: Absent.

Second instar: Absent.

Third instar: O: postero-lateral area of metathoracic dorsal region. I: postero-ventral area of metathoracic lateral region. Broad medially and narrowing towards both ends, straight.

Fourth instar: Approximate parallelogram, original end broader than insertional end.

Prepupa: Absent.

Developmental changes: Both original and insertional ends become broader in the fourth instar.

III d-v 1

First instar: Absent.

Second instar: Absent.

Third instar: Absent.

Fourth instar: Absent.

Prepupa: O: antero-lateral area of metathoracic dorsal region. I: antero-lateral area of metathoracic ventral region. Broad medially and narrowing towards both ends, bent laterad.
Developmental changes: Not applicable.

III d-v 2
First instar: Absent.
Second instar: Absent.
Third instar: Absent.
Fourth instar: Absent.
Prepupa: O: antero-lateral area of metathoracic dorsal region. I: antero-lateral area of metathoracic ventral region. Broad medially and narrowing towards both ends, bent laterad.
Developmental changes: Not applicable.

III d-v 3
First instar: Absent.
Second instar: Absent.
Third instar: Absent.
Fourth instar: Absent.
Prepupa: O: postero-lateral area of metathoracic dorsal region. I: postero-lateral area of metathoracic ventral region. Approximate parallelogram, both original and insertional ends equal, slightly bent antero-laterad.
Developmental changes: Not applicable.

III l-l 1
First instar: Absent.
Second instar: O: antero-dorsal area of metathoracic lateral region. I: postero-dorsal area of metathoracic region. Long triangle, narrowing postero-dorsad, straight.
Third instar: Bent proximad.
Fourth instar: Approximate parallelogram, original end broader than insertional end, slightly bent proximad.
Prepupa: Absent.
Developmental changes: The insertional end becomes broader in the fourth instar. The muscle is straight in the second instar, and bent proximad in the third and fourth instars.

III l-l 2
First instar: Absent.
Second instar: Absent.
Third instar: O: postero-median area of metathoracic lateral region. I: postero-ventral area of metathoracic lateral region. Long triangle, narrowing towards ventro-proximad, slightly

bent ventro-laterad.

Fourth instar: Absent.

Prepupa: Long triangle, narrowing towards antero-dorsad, slightly bent antero-laterad.

Developmental changes: The original end becomes narrower in the prepupa. The insertional end becomes broader in the prepupa. The muscle is slightly bent ventro-laterad in the third instar and slightly bent antero-laterad in the prepupa.

III I-I 3

First instar: Absent.

Second instar: Absent.

Third instar: Absent.

Fourth instar: Absent.

Prepupa: O: antero-dorsal area of metathoracic lateral region. I: antero-median area of metathoracic lateral region. Parallelogram, slightly bent postero-laterad.

Developmental changes: Not applicable.

III I-v 1

First instar: Absent.

Second instar: Absent.

Third instar: Absent.

Fourth instar: Absent.

Prepupa: O: antero-dorsal area of metathoracic lateral region. I: antero-lateral area of metathoracic ventral region. Parallelogram, bent antero-laterad.

Developmental changes: Not applicable.

III I-leg 1

First instar: Absent.

Second instar: O: antero-ventral area of metathoracic lateral region. I: antero-lateral area of metathoracic ventral region. Parallelogram, slightly bent proximad.

Third instar: O: antero-median area of metathoracic lateral region. O: postero-lateral area of metathoracic ventral region. Approximate parallelogram, original end broader than insertional end, straight.

Fourth instar Almost the same as the last stage.

Prepupa: Approximate parallelogram, original end narrower than insertional end.

Developmental changes: The original end moves dorsad in the third instar. The insertional end moves posterad in the third instar. The muscle is slight bent proximad in the second instar, and straight in the third and fourth instars.

III I-leg 2

First instar: Absent.
Second instar: O: dorso-median area of metathoracic lateral region. I: antero-lateral margin of basal rim of hindleg. Long triangle, narrowing towards metathoracic lateral region, straight.
Third instar: O: antero-median area of metathoracic lateral region. I: antero-lateral margin of basal rim of hindleg. Approximate parallelogram, original end broader than insertional end.
Fourth instar: Approximate parallelogram, original end narrower than insertional end.
Prepupa: Almost the same as the last stage.
Developmental changes: The original end becomes broader in the fourth instar.

III l-leg 3
First instar: Absent.
Second instar: Absent.
Third instar: O: dorso-median area of metathoracic lateral region. I: latero-median margin of basal rim of hindleg. Approximate parallelogram, both original and insertional ends equal, slightly bent postero-proximad.
Fourth instar: O: dorso-anterior area of metathoracic lateral region. I: latero-anterior margin of basal rim of hindleg. Long triangle, narrowing towards metathoracic lateral region, curved.
Prepupa: O: dorso-anterior area of metathoracic lateral region. I: latero-posterior margin of basal rim of hindleg. Approximate parallelogram, both original and insertional ends equal, slightly bent laterad.
Developmental changes: The original end moves anterad in the fourth instar. The insertional end moves anterad in the fourth instar and posterad in the prepupa. The original end becomes narrower in the fourth instar and broader in the prepupa. The muscle is slight bent bent postero-proximad in the third instar, curved in the fourth instar and slightly bent laterad in the prepupa.

III l-leg 4
First instar: Absent.
Second instar: Absent.
Third instar: Absent.
Fourth instar: Absent.
Prepupa: O: postero-ventral area of metathoracic lateral region. I: antero-lateral margin of basal rim of hindleg. Long triangle, narrowing towards hindleg, bent antero-proximad.
Developmental changes: Not applicable.

III v-v 1
First instar: Absent.
Second instar: Absent.
Third instar: O: antero-median area of metathoracic ventral region. I: postero-median area

of metathoracic ventral region. Approximate parallelogram, original end narrower than insertional end, straight.

Fourth instar: Slightly bent dorso-laterad.

Prepupa: Absent.

Developmental changes: The muscle is straight in the third instar and slightly bent dorso-laterad in the fourth instar.

III v-v 2

First instar: Absent.

Second instar: Absent.

Third instar: O: antero-median area of metathoracic ventral region. I: postero-median area of metathoracic ventral region. Long triangle, narrowing posterad, straight.

Fourth instar: O: antero-lateral area of metathoracic ventral region. I: postero-lateral area of metathoracic ventral region. Approximate parallelogram, original end narrower than insertional end, slightly bent dorso-proximad.

Prepupa: Straight.

Developmental changes: Both original and insertional ends move laterad in the fourth instar. The muscle is straight in the third and fourth instars, and slightly bent dorso-proximad in the fourth instar.

III v-leg 1

First instar: Absent.

Second instar: O: central area of metathoracic ventral region. I: antero-proximal margin of basal rim of hindleg. Long triangle, narrowing towards metathoracic ventral region, straight.

Third instar: Approximate parallelogram, original end narrower than insertional end.

Fourth instar: O: antero-median area of metathoracic ventral region. I: postero-lateral margin of basal rim of hindleg. Slightly bent dorso-proximad.

Prepupa: Absent.

Developmental changes: The original end moves anterad in the fourth instar. The insertional end moves postero-laterad in the fourth instar. The original end becomes broader in the third instar. The muscle is straight in the second and third instars, and slightly bent dorso-proximad in the fourth instar.

III v-leg 2

First instar: Absent.

Second instar: O: antero-median area of metathoracic ventral region. I: postero-lateral margin of basal rim of hindleg. Broad medially and narrowing towards both ends, straight.

Third instar: Long triangle, narrowing towards metathoracic ventral region, slightly bent antero-laterad.

Fourth instar: O: antero-median area of metathoracic ventral region. I: antero-lateral margin of basal rim of hindleg.
Prepupa: Absent.
Developmental changes: The insertional end moves anterad in the fourth instar. The muscle is straight in the second instar and slightly bent antero-laterad in the third and fourth instars.

III leg-leg 1
First instar: Absent.
Second instar: O: antero-proximal margin of basal rim of hindleg. I: postero-proximal margin of basal rim of hindleg. Triangle, narrowing anterad, straight.
Third instar: Parallelogram.
Fourth instar: Approximate parallelogram, original end narrower than insertional end.
Prepupa: Absent.
Developmental changes: The original end becomes broader in the third instar.

III leg-leg 2
First instar: Absent.
Second instar: Absent.
Third instar: O: antero-proximal margin of basal rim of hindleg. I: postero-proximal margin of basal rim of hindleg. Approximate parallelogram, original end broader than insertional end, straight.
Fourth instar: Almost the same as the last stage.
Prepupa: Absent.
Developmental changes: None.

Intersegmental muscles

T Iv-III 1
First instar: Absent.
Second instar: Absent.
Third instar: O: postero-median area of prothoracic ventral region. I: postero-median area of mesothoracic lateral region. Broad medially and narrowing towards both ends, bent ventro-laterad.
Fourth instar: Absent.
Prepupa: Absent.
Developmental changes: Not applicable.

T Iv-IIv 1
First instar: Absent.

Second instar: O: meso-lateral area of prothoracic ventral region. I: postero-lateral area of mesothoracic ventral region. Parallelogram, slightly bent antero-dorsad.
Third instar: Absent.
Fourth instar: Absent.
Prepupa: Absent.
Developmental changes: Not applicable.

T Iv-IIv 2
First instar: Absent.
Second instar: O: antero-median area of prothoracic ventral region. I: postero-lateral area of mesothoracic ventral region. Approximate parallelogram, original end broader than insertional end, slightly bent postero-ventrad.
Third instar: Absent.
Fourth instar: Absent.
Prepupa: Absent.
Developmental changes: Not applicable.

T III-h 1
First instar: O: antero-median area of mesothoracic lateral region. I: postero-lateral area of head. Long triangle, narrowing towards head, curved.
Second instar: O: postero-median area of mesothoracic lateral region. I: postero-lateral area of head. Straight.
Third instar: O: postero-ventral area of mesothoracic lateral region. I: postero-lateral area of head. Approximate parallelogram, original end broader than insertional end.
Fourth instar: O: postero-median area of mesothoracic lateral region. I: postero-lateral area of head.
Prepupa: O: postero-dorsal area of mesothoracic lateral region. I: postero-lateral area of head. Curved.
Developmental changes: The original end moves postero-ventrad from first to third instars and dorsad in the fourth instar. The insertional end becomes broader in the third instar. The muscle is curved in the first instars and prepupa, and straight in the second to fourth instars.

T III-h 2
First instar: O: postero-ventral area of mesothoracic lateral region. I: postero-lateral area of head. Approximate parallelogram, original end narrower than insertional end, straight.
Second instar: Absent.
Third instar: O: antero-ventral area of mesothoracic lateral region. I: postero-lateral area of head. Slightly bent postero-ventrad.
Fourth instar: Curved.

Prepupa: Absent.

Developmental changes: The original end moves anterad in the third instar. The muscle is straight in the first instar, slightly bent postero-ventrad in the third instar and curved in the fourth instar.

T IId-IIId 1

First instar: Absent.

Second instar: O: antero-median area of mesothoracic dorsal region. I: postero-lateral area of metathoracic dorsal region. Approximate parallelogram, original end narrower than insertional end, slightly bent dorso-posterad.

Third instar: Absent.

Fourth instar: Absent.

Prepupa: Absent.

Developmental changes: Not applicable.

T IId-IIIl 1

First instar: Absent.

Second instar: O: antero-lateral area of mesothoracic dorsal region. I: postero-dorsal area of metathoracic lateral region. Approximate Parallelogram, original end narrower than insertional end, slightly bent dorso-laterad.

Third instar: Absent.

Fourth instar: Absent.

Prepupa: Absent.

Developmental changes: Not applicable.

T IId-IIIv 1

First instar: Absent.

Second instar: O: meso-lateral area of mesothoracic dorsal region. I: antero-lateral area of metathoracic ventral region. Broad medially and narrowing towards both ends, slightly bent laterad.

Third instar: Absent.

Fourth instar: Absent.

Prepupa: Absent.

Developmental changes: Not applicable.

T III-IIIv 1

First instar: Absent.

Second instar: O: antero-ventral area of mesothoracic lateral region. I: antero-lateral area of metathoracic ventral region. Broad medially and narrowing towards both ends, slightly bent

latero-ventrad.
Third instar: Absent.
Fourth instar: Absent.
Prepupa: Absent.
Developmental changes: Not applicable.

T IIv-h 1
First instar: O: postero-lateral area of mesothoracic ventral region. I: postero-ventral area of lateral side of head. Long triangle, narrowing towards head, slightly bent dorsad.
Second instar: O: antero-lateral area of mesothoracic ventral region. I: postero-median area of lateral side of head. Approximate parallelogram, both original and insertional ends equal, slightly bent postero-laterad.
Third instar: Almost the same as the last stage.
Fourth instar: Almost the same as the last stage.
Prepupa: O: antero-lateral area of mesothoracic ventral region. I: postero-ventral area of lateral side of head. Bent postero-proximad.
Developmental changes: The original end moves anterad in the second instar. The insertional end moves dorsad in the second instar and ventrad in the prepupa. The muscle is slightly bent dorsad in the first instar, slightly bent postero-laterad in the second to fourth instars and bent postero-proximad in the prepupa.

T IIv-IIIl 1
First instar: Absent.
Second instar: Absent.
Third instar: O: postero-lateral area of mesothoracic ventral region. I: antero-ventral area of metathoracic lateral region. Broad medially and narrowing towards both ends, straight.
Fourth instar: Absent.
Prepupa: Absent.
Developmental changes: Not applicable.

T IIv-IIIv 1
First instar: Absent.
Second instar: O: antero-median area of mesothoracic ventral region. I: antero-lateral area of metathoracic ventral region. Approximate parallelogram, original end broader than insertional end, straight.
Third instar: O: antero-median area of mesothoracic ventral region. I: meso-lateral area of metathoracic ventral region. Approximate parallelogram, original end narrower than insertional end.
Fourth instar: Absent.

Prepupa: Absent.
Developmental changes: The insertional end moves posterad in the third instar.

T IIId-At 1
First instar: Absent.
Second instar: O: antero-lateral area of metathoracic dorsal region. I: latero-median area of first abdominal dorsal region. Approximate parallelogram, original end broader than insertional end, slightly bent dorso-laterad.
Third instar: Absent.
Fourth instar: Absent.
Prepupa: Absent.
Developmental changes: Not applicable.

T IIIl-h 1
First instar: Absent.
Second instar: O: postero-dorsal area of metathoracic lateral region. I: ventro-median area of lateral side of head. Broad medially and narrowing towards both ends, slightly bent ventro-posterad.
Third instar: Long triangle, narrowing towards head.
Fourth instar: Almost the same as the last stage.
Prepupa: Curved.
Developmental changes: The original end becomes broader in the third instar. The muscle is slightly bent ventro-posterad in the second and third and fourth instars, and curved in the prepupa.

T IIIv-h 1
First instar: Absent.
Second instar: O: antero-lateral area of metathoracic ventral region. I: ventro-median area of lateral side of head. Approximate parallelogram, original end broader than insertional end, straight.
Third instar: Almost the same as the last stage.
Fourth instar: Slightly bent latero-ventrad.
Prepupa: Slightly bent dorsad.
Developmental changes: The muscle is straight in the second and third instars, slightly bent latero-ventrad in the fourth instar, and slightly bent dorsad in the prepupa.
