# Supplementary material for: Transformation of Internal Thoracic Structures of Callobruchus maculatus (Coleoptera: Bruchidae) from Larva to Adult
Source: Insects. 2025 Mar 19;16(3):324. doi: 10.3390/insects16030324 (PMC11943184; doi:10.3390/insects16030324)
Supplement: Supplementary file 1 [file insects-16-00324-s001.zip › S3 Thoracic measurement of larvae.pdf]

**Supplementary Material S3: Thoracic length, width and height, muscular absolute and relative volumes of the larvae**

**Table S1. The absolute volumes ($\mu m^3$) of thoracic muscles in each larval developmental stage. The absolute volume larger than that in the last developmental stage is denoted in blue, while shorter length is denoted in yellow. Absent is denoted with "-".**

| | 1st instar | 2nd instar | 3rd instar | 4th instar | Prepupa |
|---|---|---|---|---|---|
| Prothorax | | | | | |
| I dam-h 1 | 9640.95 | 40989.04 | 149299.18 | - | - |
| I dam-h 2 | - | 48082.56 | 63603.89 | 1239361.07 | - |
| I dal-h 1 | 10471.67 | 113658.80 | 155884.82 | 225134.78 | 109032.85 |
| I dal-h 2 | 9783.64 | 58669.30 | 107622.86 | 131669.80 | - |
| I dal-h 3 | - | 68734.29 | 60941.78 | 84566.97 | 471178.65 |
| I dal-h 4 | - | 52646.88 | 120704.05 | 108966.08 | 172636.11 |
| I dml-h 1 | 3882.14 | 51755.44 | 128362.73 | 287400.72 | - |
| I dml-h 2 | 12865.89 | 95940.54 | 249550.05 | - | - |
| I dml-h 3 | - | 24196.77 | 71737.65 | - | - |
| I dml-h 4 | - | 17531.68 | 149585.87 | - | - |
| I dml-h 5 | - | 71052.73 | - | 320707.19 | - |
| I dpl-h 1 | 16273.41 | 107180.30 | 138462.35 | 520348.93 | 209102.46 |
| I dpl-h 2 | 12212.32 | 94817.60 | 211297.62 | 312547.46 | 196870.72 |
| I dpl-h 3 | 8364.04 | 120120.03 | 186372.10 | 314014.68 | 184391.77 |
| I dpl-h 4 | - | 108334.34 | 148365.39 | 180987.02 | 172379.75 |
| I dpl-h 5 | - | 17466.04 | 98014.70 | 741582.12 | 325890.02 |
| I dpl-h 6 | - | 61516.38 | - | 188126.08 | 186506.69 |
| I dpm-h 1 | 6971.64 | 99295.54 | 168777.62 | 282275.68 | 105517.14 |
| I d-d 1 | 3518.77 | - | - | - | - |
| I l-h 1 | 6344.07 | 44240.38 | 124504.72 | 171841.92 | 360552.75 |
| I l-h 2 | 16839.92 | 83716.04 | 139011.16 | - | 188539.21 |
| I l-h 3 | 18853.85 | 44938.33 | 75390.88 | 167738.70 | - |
| I l-h 4 | - | 176764.58 | - | - | - |
| I l-l 1 | - | 23474.63 | 60499.46 | - | - |
| I l-l 2 | - | 130889.89 | 169121.64 | 522783.29 | 159598.68 |
| I l-l 3 | - | 41410.57 | 69337.66 | - | 164551.80 |
| I l-leg 1 | 7943.23 | 65427.67 | 84442.05 | - | - |
| I v-h 1 | - | 43176.18 | 121883.57 | 84694.67 | - |
| I v-h 2 | - | - | 76472.11 | 156022.55 | - |
| I leg-h 1 | 9867.07 | 30668.36 | 92600.39 | 325111.14 | - |
| I leg-h 2 | 23641.67 | 81397.60 | 165394.69 | 433219.90 | - |
| I leg-v 1 | - | 26155.86 | 123628.27 | 237398.87 | 222680.07 |
| I leg-v 2 | - | - | - | 118713.10 | - |

| | | | | | |
|---|---|---|---|---|---|
| **I leg-v 3** | - | - | - | 558762.21 | - |
| **I leg-v 4** | - | - | - | 208932.65 | - |
| **I leg-leg 1** | 9970.46 | 41783.73 | 48450.36 | 148910.83 | - |
| **Mesothorax** | | | | | |
| **II d-d 1** | - | 75630.87 | - | 1079174.93 | - |
| **II d-d 2** | - | 55725.47 | 78356.06 | 402520.95 | - |
| **II d-d 3** | - | 50978.02 | 66126.75 | 147442.47 | 173505.88 |
| **II d-l 1** | - | 107916.26 | - | - | - |
| **II l-l 1** | - | 48262.23 | 195300.40 | 238191.72 | - |
| **II l-l 2** | - | 68665.19 | 154926.47 | - | - |
| **II l-l 3** | - | 73910.18 | 197552.95 | 276559.43 | - |
| **II l-l 4** | - | - | 54741.12 | - | - |
| **II l-l 5** | - | - | 92584.00 | - | - |
| **II l-l 6** | - | - | 253014.89 | 390741.00 | - |
| **II l-v 1** | - | 69269.85 | 111284.28 | 253354.94 | 273950.87 |
| **II l-v 2** | - | 108455.27 | - | - | - |
| **II l-v 3** | - | - | 158972.87 | 357232.90 | 98458.25 |
| **II l-leg 1** | - | 84538.38 | 104248.13 | - | 144263.21 |
| **II l-leg 2** | - | - | - | - | 156192.83 |
| **II l-leg 3** | - | - | - | - | 314491.43 |
| **II v-v 1** | - | 33180.29 | 142713.54 | 193597.41 | 220821.50 |
| **II v-v 2** | - | 22731.76 | 48638.75 | - | 209917.30 |
| **II v-v 3** | - | - | - | 361126.51 | - |
| **II leg-v 1** | - | - | - | 273979.26 | - |
| **II leg-leg 1** | - | 66236.18 | 154287.56 | 255415.67 | - |
| **II leg-leg 2** | - | - | 101209.23 | 314579.70 | - |
| **II leg-leg 3** | - | - | - | 76069.25 | - |
| **Metathorax** | | | | | |
| **III d-d 1** | - | 99706.71 | 186765.27 | 1348127.12 | - |
| **III d-d 2** | - | 24687.40 | - | 1150963.03 | - |
| **III d-d 3** | - | 59540.01 | 173987.16 | 519537.86 | 276688.37 |
| **III d-d 4** | - | 14612.04 | 199420.52 | 488756.88 | 407868.37 |
| **III d-d 5** | - | 100985.13 | 344140.88 | 656641.40 | 117831.29 |
| **III d-l 1** | - | 120741.96 | 257536.38 | 434925.21 | 189784.36 |
| **III d-l 2** | - | - | 145678.71 | 267988.46 | - |
| **III d-v 1** | - | - | - | - | 565736.66 |
| **III d-v 2** | - | - | - | - | 956319.44 |
| **III d-v 3** | - | - | - | - | 597890.77 |
| **III l-l 1** | - | 54605.98 | 186838.99 | 178397.73 | - |
| **III l-l 2** | - | - | 18783.02 | - | 539561.08 |
| **III l-l 3** | - | - | - | - | 124212.67 |
| **III l-v 1** | - | - | - | - | 311891.26 |
| **III l-leg 1** | - | 63482.39 | 423758.37 | 775208.69 | 354299.54 |

| | | | | | |
|---|---|---|---|---|---|
| **III l-leg 2** | - | 85471.28 | 158186.52 | 146676.96 | 770774.08 |
| **III l-leg 3** | - | - | 104575.77 | 449924.40 | 346261.01 |
| **III l-leg 4** | - | - | - | - | 214266.17 |
| **III v-v 1** | - | - | 237672.96 | 511742.65 | - |
| **III v-v 2** | - | - | 50178.68 | 293590.86 | 472835.80 |
| **III v-leg 1** | - | 27005.84 | 61547.93 | 173401.42 | - |
| **III v-leg 2** | - | 120952.73 | 307846.11 | 186138.26 | - |
| **III leg-leg 1** | - | 43424.95 | 215032.76 | 425479.38 | - |
| **III leg-leg 2** | - | - | 119139.55 | 351708.02 | - |
| **Intersegmental muscles** | | | | | |
| **T Iv-III 1** | - | - | 159284.13 | - | - |
| **T Iv-IIv 1** | - | 44782.85 | - | - | - |
| **T Iv-IIv 2** | - | 120952.73 | - | - | - |
| **T IId-IIId 1** | - | 65724.81 | - | - | - |
| **T IId-IIIl 1** | - | 108006.10 | - | - | - |
| **T IId-IIIv 1** | - | 152833.86 | - | - | - |
| **T III-h 1** | 10683.89 | 136055.41 | 262451.04 | 615966.91 | 298698.19 |
| **T III-h 2** | 17029.77 | - | 83049.56 | 250573.14 | - |
| **T III-IIIv 1** | - | 129017.17 | - | - | - |
| **T IIv-h 1** | 9700.20 | 146794.17 | 280012.75 | 401654.05 | 136362.02 |
| **T IIv-IIIl 1** | - | - | 74866.65 | - | - |
| **T IIv-IIIv 1** | - | 76971.49 | 380419.26 | - | - |
| **T IIId-At 1** | - | 140312.27 | - | - | - |
| **T IIIl-h 1** | - | 60987.74 | 247993.74 | 318846.96 | 150150.2 |
| **T IIIv-h 1** | - | 250695.49 | 527990.11 | 1426409.51 | 623123.33 |

**Table S2. The relative volumes (= absolute volume * $10^6$ / (length * width * height)) of thoracic muscles in each larval developmental stage. The relative volume larger than that in the last developmental stage is denoted in blue, while shorter length is denoted in yellow. Absent is denoted with "-".**

| | 1st instar | 2nd instar | 3rd instar | 4th instar | Prepupa |
|---|---|---|---|---|---|
| **Prothorax** | | | | | |
| **I dam-h 1** | 429.51 | 142.85 | 178.16 | - | - |
| **I dam-h 2** | - | 167.57 | 75.90 | 574.05 | - |
| **I dal-h 1** | 466.52 | 396.11 | 186.02 | 104.28 | 43.22 |
| **I dal-h 2** | 435.86 | 204.47 | 128.43 | 60.99 | - |
| **I dal-h 3** | - | 239.54 | 72.72 | 39.17 | 186.79 |
| **I dal-h 4** | - | 183.48 | 144.04 | 50.47 | 68.44 |
| **I dml-h 1** | 172.95 | 180.37 | 153.18 | 133.12 | - |
| **I dml-h 2** | 573.18 | 334.36 | 297.80 | - | - |
| **I dml-h 3** | - | 84.33 | 85.61 | - | - |

| | | | | | |
|---|---|---|---|---|---|
| **I dml-h 4** | - | 61.10 | 178.51 | - | - |
| **I dml-h 5** | - | 247.62 | - | 148.55 | - |
| **I dpl-h 1** | 724.99 | 373.53 | 165.23 | 241.02 | 82.90 |
| **I dpl-h 2** | 544.06 | 330.45 | 252.15 | 144.77 | 78.05 |
| **I dpl-h 3** | 372.62 | 418.63 | 222.40 | 145.45 | 73.10 |
| **I dpl-h 4** | - | 377.55 | 177.05 | 83.83 | 68.34 |
| **I dpl-h 5** | - | 60.87 | 116.96 | 343.49 | 129.19 |
| **I dpl-h 6** | - | 214.39 | - | 87.14 | 73.94 |
| **I dpm-h 1** | 310.59 | 346.05 | 201.41 | 130.74 | 41.83 |
| **I d-d 1** | 156.76 | - | - | - | - |
| **I l-h 1** | 282.63 | 154.18 | 148.58 | 79.59 | 142.94 |
| **I l-h 2** | 750.22 | 291.76 | 165.89 | - | 74.74 |
| **I l-h 3** | 839.94 | 156.61 | 89.97 | 77.69 | - |
| **I l-h 4** | - | 616.04 | - | - | - |
| **I l-l 1** | - | 81.81 | 72.20 | - | - |
| **I l-l 2** | - | 456.16 | 201.82 | 242.14 | 63.27 |
| **I l-l 3** | - | 144.32 | 82.74 | - | 65.23 |
| **I l-leg 1** | 353.87 | 228.02 | 100.77 | - | - |
| **I v-h 1** | - | 150.47 | 145.45 | 39.23 | - |
| **I v-h 2** | - | - | 91.26 | 72.27 | - |
| **I leg-h 1** | 439.58 | 106.88 | 110.50 | 150.59 | - |
| **I leg-h 2** | 1053.24 | 283.68 | 197.37 | 200.66 | - |
| **I leg-v 1** | - | 91.16 | 147.53 | 109.96 | 88.28 |
| **I leg-v 2** | - | - | - | 54.99 | - |
| **I leg-v 3** | - | - | - | 258.81 | - |
| **I leg-v 4** | - | - | - | 96.77 | - |
| **I leg-leg 1** | 444.19 | 145.62 | 57.82 | 68.97 | - |
| **Mesothorax** | | | | | |
| **II d-d 1** | - | 263.58 | - | 499.85 | - |
| **II d-d 2** | - | 194.21 | 93.51 | 186.44 | - |
| **II d-d 3** | - | 177.66 | 78.91 | 68.29 | 68.78 |
| **II d-l 1** | - | 376.10 | - | - | - |
| **II l-l 1** | - | 168.20 | 233.06 | 110.33 | - |
| **II l-l 2** | - | 239.30 | 184.88 | - | - |
| **II l-l 3** | - | 257.58 | 235.75 | 128.10 | - |
| **II l-l 4** | - | - | 65.32 | - | - |
| **II l-l 5** | - | - | 110.48 | - | - |
| **II l-l 6** | - | - | 301.93 | 180.98 | - |
| **II l-v 1** | - | 241.41 | 132.80 | 117.35 | 108.60 |
| **II l-v 2** | - | 377.98 | - | - | - |
| **II l-v 3** | - | - | 189.71 | 165.46 | 39.03 |
| **II l-leg 1** | - | 294.62 | 124.40 | - | 57.19 |
| **II l-leg 2** | - | - | - | - | 61.92 |

| | | | | | |
|---|---|---|---|---|---|
| **II l-leg 3** | - | - | - | - | 124.68 |
| **II v-v 1** | - | 115.64 | 170.31 | 89.67 | 87.54 |
| **II v-v 2** | - | 79.22 | 58.04 | - | 83.22 |
| **II v-v 3** | - | - | - | 167.27 | - |
| **II leg-v 1** | - | - | - | 126.90 | - |
| **II leg-leg 1** | - | 230.84 | 184.12 | 118.30 | - |
| **II leg-leg 2** | - | - | 120.78 | 145.71 | - |
| **II leg-leg 3** | - | - | - | 35.23 | - |
| **Metathorax** | | | | | |
| **III d-d 1** | - | 347.49 | 222.87 | 624.43 | - |
| **III d-d 2** | - | 86.04 | - | 533.10 | - |
| **III d-d 3** | - | 207.50 | 207.63 | 240.64 | 109.69 |
| **III d-d 4** | - | 50.92 | 237.98 | 226.38 | 161.69 |
| **III d-d 5** | - | 351.94 | 410.68 | 304.14 | 46.71 |
| **III d-l 1** | - | 420.80 | 307.33 | 201.45 | 75.24 |
| **III d-l 2** | - | - | 173.84 | 124.13 | - |
| **III d-v 1** | - | - | - | - | 224.28 |
| **III d-v 2** | - | - | - | - | 379.12 |
| **III d-v 3** | - | - | - | - | 237.03 |
| **III l-l 1** | - | 190.31 | 222.96 | 82.63 | - |
| **III l-l 2** | - | - | 220.51 | - | 213.90 |
| **III l-l 3** | - | - | - | - | 49.24 |
| **III l-v 1** | - | - | - | - | 123.65 |
| **III l-leg 1** | - | 221.24 | 505.69 | 359.06 | 140.46 |
| **III l-leg 2** | - | 297.87 | 188.77 | 67.94 | 305.56 |
| **III l-leg 3** | - | - | 124.79 | 208.40 | 137.27 |
| **III l-leg 4** | - | - | - | - | 84.94 |
| **III v-v 1** | - | - | 283.62 | 237.03 | - |
| **III v-v 2** | - | - | 59.88 | 135.99 | 187.45 |
| **III v-leg 1** | - | 94.12 | 73.45 | 80.32 | - |
| **III v-leg 2** | - | 421.53 | 367.36 | 86.22 | - |
| **III leg-leg 1** | - | 151.34 | 256.61 | 197.07 | - |
| **III leg-leg 2** | - | - | 142.17 | 162.90 | - |
| **Intersegmental muscles** | | | | | |
| **T Iv-III 1** | - | - | 190.08 | - | - |
| **T Iv-IIv 1** | - | 156.07 | - | - | - |
| **T Iv-IIv 2** | - | 421.53 | - | - | - |
| **T IId-IIId 1** | - | 229.06 | - | - | - |
| **T IId-IIIl 1** | - | 376.41 | - | - | - |
| **T IId-IIIv 1** | - | 532.64 | - | - | - |
| **T III-h 1** | 475.97 | 474.16 | 313.19 | 285.30 | 118.42 |
| **T III-h 2** | 758.68 | - | 99.11 | 116.06 | - |
| **T III-IIIv 1** | - | 449.63 | - | - | - |

| | | | | | |
|---|---|---|---|---|---|
| **T IIv-h 1** | 432.15 | 511.59 | 344.15 | 186.04 | 54.06 |
| **T IIv-IIIl 1** | - | - | 89.34 | - | - |
| **T IIv-IIIv 1** | - | 268.25 | 453.97 | - | - |
| **T IIId-At 1** | - | 489.00 | - | - | - |
| **T IIIl-h 1** | - | 212.55 | 295.94 | 147.68 | 59.53 |
| **T IIIv-h 1** | - | 873.69 | 630.07 | 660.69 | 247.03 |
