# Supplementary material for: Transformation of Internal Thoracic Structures of Callobruchus maculatus (Coleoptera: Bruchidae) from Larva to Adult
Source: Insects. 2025 Mar 19;16(3):324. doi: 10.3390/insects16030324 (PMC11943184; doi:10.3390/insects16030324)
Supplement: Supplementary file 1 [file insects-16-00324-s001.zip › S4 Pupal and adult muscle description.pdf]

Supplementary Material S4: Pupal and adult musculature

Prothoracic muscles

Idlm1 M. prophragma-occipitalis

Initial pupal stage: Absent.

Middle pupal stage: O: dorso-lateral area of intersegmental ridge between pro- and mesonotum. I: dorso-median area of occiput. Approximate parallelogram, original end broader than insertional end, slightly bent ventrad.

Late pupal stage: O: latero-median area of prophragma. I: dorso-median area of occiput. Slightly bent dorsad.

Adult: O: later-median area of prophragma. I: dorso-median area of occiput. Slightly bent ventad.

Developmental changes: The original site changes from intersegmental ridge to the prophragma. The muscle is slightly bent ventrad in the middle pupal stage and adult, and slightly bent dorsad in the late pupal stage.

Idlm2 M. pronoto-occipitalis

Initial pupal stage: Absent.

Middle pupal stage: O: postero-median area of pronotum. I: dorso-lateral area of occiput. Narrow medially and broadening towards both ends, straight.

Late pupal stage: Triangle, narrowing towards occiput.

Adult: Long triangle, narrowing towards occiput.

Developmental changes: The insertional end becomes narrower in the late pupal stage.

Idlm3 M. prophragma-cervicalis

Initial pupal stage: Absent.

Middle pupal stage: Absent.

Late pupal stage: O: lateral area of intersegmental ridge between pro- and mesonotum. I: cervical membrane. Broad medially and narrowing towards both ends, slightly bent dorsad.

Adult: O: dorso-lateral area of prophragma. I: cervical membrane. Long triangle, narrowing towards prophragma, curved.

Developmental changes: The original site changes from intersegmental ridge to the prophragma. The insertional end becomes broader in the adult. The muscle is slightly bent dorsad in the late pupal stage and curved in the adult.

Idlm5 M. pronoto-phragmalis anterior

Initial pupal stage: Absent.

Middle pupal stage: O: antero-median area of pronotum. I: latero-ventral area of intersegmental ridge of pro- and mesonotum. Approximate parallelogram, original end broader than insertional end, slightly bent latero-dorsad.
Late pupal stage: Straight.
Adult: O: antero-median area of pronotum. I: latero-ventral area of prophragma. Slightly bent latero-dorsad.
Developmental changes: The insertional site changes from intersegmental ridge to the pronotum in the adult. The muscle is slightly bent latero-dorsad in the middle pupal stage and adult, and straight in the late pupal stage.

Idlm6 M. pronoto-phragmalis posterior
Initial pupal stage: Absent.
Middle pupal stage: Absent.
Late pupal stage: O: postero-lateral area of pronotum. I: lateral area of prophragma.
Adult: Absent.
Developmental changes: Not applicable.

Idvm2 M. cervico-occipitalis medialis
Initial pupal stage: Absent.
Middle pupal stage: Absent.
Late pupal stage: O: antero-lateral area of pronotum. I: antero-median area of prosternum. Approximate parallelogram, original end narrower than insertional end, bent latero-posterad.
Adult: Almost the same as the last stage.
Developmental changes: None.

Idvm6 M. pronoto-cervicalis medialis
Initial pupal stage: Absent.
Middle pupal stage: Absent.
Late pupal stage: O: latero-median area of pronotum. I: cervical membrane. Approximate parallelogram, original end broader than insertional end, straight.
Adult: Slightly bent laterad.
Developmental changes: The muscle is straight in the late pupal stage and slightly bent laterad in the adult.

Idvm7 M. pronoto-cervicalis posterior
Initial pupal stage: Absent.
Middle pupal stage: O: postero-lateral area of pronotum. I: cervical membrane. Long triangle, narrowing towards cervical membrane, bent antero-laterad.
Late pupal stage: Straight.

Adult: Almost the same as the last stage.

Developmental changes: The muscle is bent antero-laterad in the middle pupal stage, and straight in the late pupal stage and adult.

Idvm8 M. prophragma-tentorialis

Initial pupal stage: Absent.

Middle pupal stage: Absent.

Late pupal stage: O: dorso-median area of prophragma. I: dorso-lateral area of occiput. Approximate parallelogram, original end narrower than insertional end, slightly bent dorsad.

Adult: Broad medially and narrowing towards both ends, slightly bent ventrad.

Developmental changes: Both original and insertional ends become narrower in the adult. The muscle is slightly bent dorsad in the late pupal stage and bent ventrad in the adult.

Idvm9 M. profurca-occipitalis

Initial pupal stage: Absent.

Middle pupal stage: Absent.

Late pupal stage: Absent.

Adult: O: anterior face of apex of profurcal arm. I: ventro-median area of occiput. Broad medially and narrowing towards both ends, straight.

Developmental changes: Not applicable.

Idvm10 M. profurca-phragmalis

Initial pupal stage: Absent.

Middle pupal stage: Absent.

Late pupal stage: Absent.

Adult: O: median area of proximal face of profurcal arm. I: latero-ventral area of prophragma. Broad medially and narrowing towards both ends, straight.

Developmental changes: Not applicable.

Idvm18 M. pronoto-coxalis lateralis

Initial pupal stage: Absent.

Middle pupal stage: O: postero-lateral area of pronotum. I: extends to procoxa. Long triangle, narrowing towards procoxa, bent postero-laterad

Late pupal stage: O: postero-lateral area of pronotum. I: postero-lateral margin of procoxal rim.

Adult: Almost the same as the last stage.

Developmental changes: The insertional site extends ventrad until it connects with the procoxal rim in the late pupal stage.

Itpm3 M. pronoto-pleuralis anterior

Initial pupal stage: Absent.

Middle pupal stage: O: meso-lateral area of pronotum. I: crytopleural apex. Broad medially and narrowing towards both ends, straight.

Late pupal stage: O: meso-lateral area of pronotum. I: lateral area of crytopleural apex. Approximate parallelogram, both original and insertional ends equal, straight.

Adult: Almost the same as the last stage.

Developmental changes: Both original and insertional ends become broader in the late pupal stage.

Itpm4 M. pronoto-apodemalis anterior

Initial pupal stage: Absent.

Middle pupal stage: Absent.

Late pupal stage: Absent.

Adult: O: postero-lateral area of pronotum. I: postero-dorsal margin of pronotum. Triangle, narrowing antero-dorsad.

Developmental changes: Not applicable.

Itpm5 M. pronoto-apodemalis posterior

Initial pupal stage: Absent.

Middle pupal stage: O: postero-lateral area of pronotum. I: dorsal area of intersegmental ridge between pronotum and mesopleuron. Long triangle, narrowing towards intersegmental ridge, straight.

Late pupal stage: Almost the same as the last stage.

Adult: Almost the same as the last stage.

Developmental changes: None.

Itpm6 M. pronoto-intersegmentalis

Initial pupal stage: Absent.

Middle pupal stage: O: postero-lateral area of pronotum. I: dorsal area of intersegmental ridge between pronotum and mesopleuron. Long triangle, narrowing towards intersegmental ridge, slightly bent laterad.

Late pupal stage: Almost the same as the last stage.

Adult: Almost the same as the last stage.

Developmental changes: None.

Ipcm6 M. propleuro-coxalis posterior

Initial pupal stage: Absent.

Middle pupal stage: O: dorso-proximal area of cryptopleuron. I: extends towards

protrochaner. Approximate triangle, narrowing towards protrochaner, curved,

Late pupal stage: Bent laterad.

Adult: O: dorso-proximal area of cryptopleuron. I: protrochaner. Straight.

Developmental changes: The insertional end extends postero-ventrad until it connects with the protrochanter in the adult.

Ivlm1 M. profurca-cervicalis

Initial pupal stage: Absent.

Middle pupal stage: Absent.

Late pupal stage: O: antero-dorsal area of profurcal arm. I: ventro-lateral area of cervical membrane. Parallelogram, slightly bent postero-ventrad.

Adult: Slightly bent antero-dorsad.

Developmental changes: The muscle is slightly bent postero-ventrad in the late pupal stage and slightly bent antero-dorsad in the adult.

Ivlm3 M. profurca-tentorialis

Initial pupal stage: Absent.

Middle pupal stage: Absent.

Late pupal stage: O: antero-dorsal area of profurcal arm. I: postero-lateral area of occiput. Approximate parallelogram, original end broader than insertional end, slightly bent postero-ventrad.

Adult: Long triangle, narrowing towards occiput, bent antero-dorsad.

Developmental changes: The insertional end becomes narrower in the adult. The muscle is slightly bent postero-ventrad in the late pupal stage and bent antero-dorsad in the adult.

Ivlm7 M. profurca-mesofurcalis

Initial pupal stage: Absent.

Middle pupal stage: O: postero-dorsal area of profurcal arm. I: antero-median area of mesofurcal arm. Approximate parallelogram, both original and insertional end equal, straight.

Late pupal stage: Almost the same as the last stage.

Adult: Almost the same as the last stage.

Developmental changes: None.

Iscm4 M. profurca-coxalis lateralis

Initial pupal stage: Absent.

Middle pupal stage: Absent.

Late pupal stage: O: latero-dorsal area of profurcal arm. I: extends towards procoxa. Long triangle, narrowing towards procoxa, slightly bent dorso-laterad.

Adult: O: latero-dorsal area of profurcal arm. I: postero-lateral margin of procoxal rim.

Slightly bent laterad.

Developmental changes: The insertional end extends ventrad until it connects with the procoxal rim. It is slightly bent dorso-laterad in the late pupal stage and slightly bent laterad in the adult.

Mesothoracic muscles

IIdlm1 M. prophragma-mesophragmalis

Initial pupal stage: Absent.

Middle pupal stage: O: extends anterad. I: antero-median area of intersegmental ridge between meso- and metanotum. Approximate parallelogram, original end broader than insertional end, straight.

Late pupal stage: O: latero-ventral area of posterior face of prophragma. I: latero-dorsal area of anterior face of mesophragma.

Adult: Almost the same as the last stage.

Developmental changes: The original end extends anterad until it connects with the prophragma in the late pupal stage. The insertional end changes from intersegmental ridge to the mesophragma in the late pupal stage.

IIdlm2 M. mesonoto-phragmalis

Initial pupal stage: Absent.

Middle pupal stage: O: antero-lateral area of mesonotum. I: antero-lateral area of intersegmental ridge between meso- and metanotum. Long triangle, narrowing towards mesonotum, bent antero-laterad.

Late pupal stage: O: antero-lateral area of mesonotum. I: latero-ventral area of anterior face of mesophragma. Approximate long triangle, narrowing towards mesophragma, straight.

Adult: Almost the same as the last stage.

Developmental changes: The insertional end changes from the intersegmental ridge to the mesophragma in the late pupal stage. The original end becomes narrower in the late pupal stage. The insertional end becomes broader in the late pupal stage.

IIdvm1 M. mesonoto-sternalis

Initial pupal stage: Absent.

Middle pupal stage: O: latero-median area of mesonotum. I: antero-ventral area of mesopleuron. Broad medially and narrowing towards both ends, slightly curved.

Late pupal stage: Long triangle, narrowing towards mesopleuron, bent antero-laterad.

Adult: Almost the same as the last stage.

Developmental changes: The insertional end becomes broader in the late pupal stage. The muscle is slightly curved in the middle pupal stage and bent antero-laterad in the late pupal

stage and adult.

IIdvm2 M. mesonoto-trochantinalis anterior

Initial pupal stage: Absent.

Middle pupal stage: Absent.

Late pupal stage: O: latero-ventral margin of prophragma. I: antero-median margin of mesocoxal rim. Broad medially and narrowing towards both ends, straight.

Adult: Almost the same as the last stage.

Developmental changes: None.

IItpm1 M. prophragma-mesanepisternalis

Initial pupal stage: Absent.

Middle pupal stage: Absent.

Late pupal stage: O: latero-ventral area of posterior face of prophragma. I: antero-dorsal process of mesopleuron (mesobasalare). Parallelogram, straight.

Adult: Approximate parallelogram, original end narrower than insertional end.

Developmental changes: The insertional end becomes broader in the adult.

IItpm4 M. mesonoto-pleuralis anterior

Initial pupal stage: Absent.

Middle pupal stage: Absent.

Late pupal stage: O: antero-dorsal area of mesopleuron. I: dorsal area of mesopleural ridge. Approximate triangle, narrowing towards mesopleuron, straight.

Adult: Almost the same as the last stage.

Developmental changes: None.

IIspm2 M. mesofurca-pleuralis

Initial pupal stage: Absent.

Middle pupal stage: Absent.

Late pupal stage: O: dorso-lateral area of mesofurcal arm. I: antero-dorsal area of mesopleuron. Parallelogram, straight.

Adult: Almost the same as the last stage.

Developmental changes: None.

IIspm7 M. mesofurca-intersegmentalis anterior

Initial pupal stage: Absent.

Middle pupal stage: Absent.

Late pupal stage: O: dorso-lateral area of mesofurcal arm. I: latero-ventral margin of intersegmental ridge between pro- and mesonotum (behind the prophragma). Long triangle,

narrowing towards mesofurca, straight.

Adult: Almost the same as the last stage.

Developmental changes: None.

IIpcm4 M. mesanepisterno-coxalis posterior

Initial pupal stage: Absent.

Middle pupal stage: O: antero-dorsal area of mesopleuron. I: lateral margin of mesocoxal rim. Broad medially and narrowing towards both ends, slightly bent dorso-posterad.

Late pupal stage: Approximate long triangle, narrowing towards mesocoxa, slightly bent laterad.

Adult: Almost the same as the last stage.

Developmental changes: The original end becomes broader in the late pupal stage. The muscle is slightly bent dorso-posterad in the middle pupal stage and slightly bent laterad in the late pupal stage and adult.

IIvlm3 M. mesofurca-metafurcalis

Initial pupal stage: Absent.

Middle pupal stage: O: postero-dorsal area of mesofurcal arm. I: antero-ventral area of metafurca. Long triangle, narrowing toward metafurca, straight.

Late pupal stage: Almost the same as the last stage.

Adult: Almost the same as the last stage.

Developmental changes: None.

IIscm1 M. mesofurca-coxalis anterior

Initial pupal stage: Absent.

Middle pupal stage: Absent.

Late pupal stage: O: ventro-proximal area of mesofurcal arm. I: antero-lateral margin of mesocoxal rim. Long triangle, narrowing towards mesocoxa, straight.

Adult: Almost the same as the last stage.

Developmental changes: None.

IIscm2 M. mesofurca-coxalis posterior

Initial pupal stage: Absent.

Middle pupal stage: O: ventro-proximal area of mesofurcal arm. I: postero-lateral margin of mesocoxal rim. Approximate triangle, narrowing towards mesocoxa, straight.

Late pupal stage: Almost the same as the last stage.

Adult: Almost the same as the last stage.

Developmental changes: None.

IIscm6 M. mesofurca-trochanteralis

Initial pupal stage: Absent.

Middle pupal stage: O: latero-ventral area of mesofurcal arm. I: extends towards mesotrochanter. Approximate long triangle, curved.

Late pupal stage: O: latero-ventral area of mesofurcal arm. I: mesotrochanter. Straight.

Adult: Almost the same as the last stage,

Developmental changes: The insertional end extends ventrad until it connects with the mesotrochanter in the late pupal stage. The muscle is curved in the middle pupal stage and straight in the late pupal stage and adult.

Metathoracic muscles

IIIdlm1 M. mesophragma-metaphragmlis

Initial pupal stage: Absent.

Middle pupal stage: O: postero-lateral area of intersegmental ridge between meso- and metapronotum. I: antero-lateral area of intersegmental ridge between metapronotum and first abdominal tergite. Broad medially and narrowing towards both ends, straight.

Late pupal stage: O: latero-median area of posterior face of mesophragma. I: latero-median area of anterior face of metaphragma. Approximate parallelogram, original end broader than insertional end, slightly bent postero-ventrad.

Adult: Straight.

Developmental changes: The original site changes from the intersegmental ridge to the mesophragma in the late pupal stage. The insertional site changes from the intersegmental ridge to the metaphragma in the late pupal stage. Both original and insertional ends become broader in the late pupal stage. The muscle is straight in the middle pupal stage and adult, and slightly bent postero-ventrad in the late pupal stage.

IIIdlm2 M. metanoto-phragmalis

Initial pupal stage: Absent.

Middle pupal stage: O: meso-lateral area of metanotum. I: lateral area of intersegmental ridge between metanotum and first abdominal tergite. Long triangle, narrowing towards metanotum, straight.

Late pupal stage: O: meso-lateral area of metanotum. I: latero-ventral area of anterior face of metaphragma.

Adult: Almost the same as the last stage.

Developmental changes: The insertional site changes from intersegmental ridge to the metaphragma in the late pupal stage.

IIIdvm1 M. metanoto-sternalis

Initial pupal stage: O: antero-lateral area of metanotum. I: antero-median area of metasternum. Broad medially and narrowing towards both ends, bent laterad.
Middle pupal stage: Long triangle, narrowing towards metanotum, straight.
Late pupal stage: Approximate parallelogram, original end narrower insertional end.
Adult: Almost the same as the last stage.
Developmental changes: The original end becomes broader in the middle pupal stage. The insertional end becomes broader in the late pupal stage.

IIIdvm2 M. metanoto-trochantinalis anterior
Initial pupal stage: O: latero-median area of metanotum. I: latero-median area of metasternum. Broad medially and narrowing towards both ends, bent laterad.
Middle pupal stage: Slightly bent antero-laterad.
Late pupal stage: Approximate parallelogram, both original and insertional ends equal, straight.
Adult: Almost the same as the last stage.
Developmental changes: Both original and insertional ends become broader in the late pupal stage. The muscle is bent lateral in the initial pupal stage, slightly bent antero-laterad in the middle pupal stage, and straight in the late pupal stage and adult.

IIIdvm4 M. metanoto-coxalis anterior
Initial pupal stage: O: antero-median area of metapleuron. I: postero-lateral area of metasternum. Broad medially and narrowing towards both ends, slightly bent proximad.
Middle pupal stage: O: postero-lateral area of metanotum. I: postero-lateral area of metasternum. Long triangle, narrowing towards metanotum, straight.
Late pupal stage: Approximate parallelogram, original end narrower than insertional end, slightly bent anterad.
Adult: Straight.
Developmental changes: The original end becomes broader in the late pupal stage. The insertional end becomes broader in the middle pupal stage. The muscle is slightly bent proximad in the initial pupal stage, straight in the middle pupal stage and adult, and slightly bent anterad in the late pupal stage.

IIIdvm5 M. metanoto-pleuralis medialis
Initial pupal stage: Absent.
Middle pupal stage: O: postero-lateral area of metanotum. I: proximo-median margin of metacoxal rim. Long triangle, narrowing towards metacoxa, curved.
Late pupal stage: Slightly bent postero-proximad.
Adult: Straight.
Developmental changes: The muscle is curved in the middle pupal stage, slightly bent

postero-proximad in the late pupal stage, and straight in the adult.

IIIdvm8 M. metafurca-phragmalis

Initial pupal stage: Absent.

Middle pupal stage: O: apex of metafurcal arm. I: lateral area of intersegmental ridge between metanotum and first abdominal tergite. Long triangle, narrowing towards metafurca, straight.

Late pupal stage: O: apex of metafurcal arm. I: latero-ventral margin of metaphragma. Approximate parallelogram, original end broader than insertional end.

Adult: Almost the same as the last stage.

Developmental changes: The insertional end changes from the intersegmental ridge to the metaphragma. The insertional end becomes broader in the late pupal stage.

IIItpm1 M. mesophragma-metanepisternalis

Initial pupal stage: Absent.

Middle pupal stage: O: postero-lateral area of intersegmental ridge between meso- and metanotum. I: antero-dorsal area of metapleuron. Parallelogram, straight.

Late pupal stage: O: lateral area of posterior face of mesophragma. I: antero-dorsal area of metapleuron. Broad medially and narrowing towards both ends.

Adult: Approximate parallelogram, both original and insertional ends equal.

Developmental changes: The original end changes from the intersegmental ridge to the mesophragma in the late pupal stage, Both original and insertional ends become narrower in the late pupal stage and broader in the adult.

IIItpm3 M. metanoto-basalaris

Initial pupal stage: Absent.

Middle pupal stage: O: antero-lateral area of metanotum. I: extends towards antero-dorsal area of metapleuron. Long triangle, narrowing towards metapleuron, slightly bent postero-ventrad.

Late pupal stage: O: antero-lateral area of metanotum. I: antero-dorsal area of metabasalare. Long triangle, narrowing towards metanotum, slightly bent dorso-proximad.

Adult: Approximate parallelogram, original end narrower than insertional end, straight.

Developmental changes: The insertional end connects with the metabasalare in the late pupal stage. The original end becomes narrower in the late pupal stage and broader in the adult. The insertional end becomes broader in the late pupal stage. The muscle is slightly bent postero-ventrad in the middle pupal stage, slightly bent dorso-proximad in the late pupal stage and straight in the adult.

IIItpm7 M. metaepisterno-axillaris

Initial pupal stage: Absent.

Middle pupal stage: O: antero-dorsal area of metapleuron. I: latero-median area of metanotum. Long triangle, narrowing towards metanotum, slightly bent ventro-proximad.

Late pupal stage: O: postero-dorsal area of metabasalare. I: latero-median area of metanotum. Approximate parallelogram, original end broader than insertional end, slightly bent dorso-proximad.

Adult: Original and insertional ends equal, bent antero-laterad.

Developmental changes: The original end changes from the metapleuron to the metabasalare. The insertional end becomes broader in the late pupal stage. The muscle is slightly bent ventro-proximad in the middle pupal stage, slightly bent dorso-proximad in the late pupal stage and bent antero-laterad in the adult.

IIItpm8 M. metepimero-axillaris secundus

Initial pupal stage: Absent.

Middle pupal stage: Absent.

Late pupal stage: Absent.

Adult: O: antero-dorsal area of metapleuron. I: antero-lateral area of metanotum. Long triangle, narrowing towards metanotum, straight.

Developmental changes: Not applicable.

IIItpm9 M. metepimero-axillaris tertius

Initial pupal stage: Absent.

Middle pupal stage: O: dorso-median area of metapleuron. I: latero-median area of metanotum. Approximate parallelogram, both original and insertional ends equal, slightly bent ventro-proximad.

Late pupal stage: Almost the same as the last stage.

Adult: Original end broader than insertional end, straight.

Developmental changes: The original end becomes broader in the adult. The muscle is slightly bent ventro-proximad in the middle pupal stage and straight in the adult.

IIItpm10 M. metepimeron-subalaris

Initial pupal stage: Absent.

Middle pupal stage: Absent.

Late pupal stage: Absent.

Adult: O: dorso-median area of metapleuron. I: antero-lateral area of metanotum. Approximate parallelogram, both original and insertional ends equal, straight.

Developmental changes: Not applicable.

IIIspm1 M. metapleural-sternalis

Initial pupal stage: O: antero-median area of metapleuron. I: latero-median area of metasternum. Approximate parallelogram, original end narrower than insertional end, slightly bent proximad.

Middle pupal stage: O: antero-dorsal area of metapleuron. I: latero-median area of metasternum. Straight.

Late pupal stage: O: proximal side of metabasalare. I: latero-median area of metasternum.

Adult: Almost the same as the last stage.

Developmental changes: The original end changes from the metapleuron in the initial and middle pupal stages to the metabasalare in the late pupal stage. The muscle is slightly bent proximad in the initial pupal stage and straight in the middle and pupal stages and adult.

IIIpcm3 M. metanepisterno-coxalis anterior

Initial pupal stage: O: antero-median area of metapleuron. I: latero-ventral margin of metacoxal rim. Broad medially and narrowing towards both ends, slightly bent proximad.

Middle pupal stage: O: antero-dorsal area of metapleuron. I: latero-ventral margin of metacoxal rim. Long triangle, narrowing towards metapleuron, straight.

Late pupal stage: O: posterior area of metabasalare. I: latero-ventral margin of metacoxal rim. Approximate parallelogram, both original and insertional ends equal.

Adult: Almost the same as the last stage.

Developmental changes: The original end changes from metapleuron in the initial and middle pupal stage to the metabasalare in the late pupal stage. The muscle is slightly bent proximad in the initial pupal stage and straight in the middle and late pupal stage and adult.

IIIpcm4 M. metanepisterno-coxalis posterior

Initial pupal stage: O: postero-median area of metapleuron. I: dorso-lateral margin of metacoxal rim. Approximate parallelogram, original end broader than insertional end, straight.

Middle pupal stage: O: antero-dorsal area of metapleuron. I: dorso-lateral margin of metacoxal rim. Approximate long triangle, narrowing towards metacoxal rim.

Late pupal stage: Almost the same as the last stage.

Adult: Almost the same as the last stage.

Developmental changes: The original end changes from the postero-median area to the antero-dorsal area of metapleuron in the middle pupal stage.

IIIscm1 M. metafurca-coxalis anterior

Initial pupal stage: O: latero-basal area of metafurca. I: antero-ventral margin of metacoxal rim. Approximate parallelogram, both original and insertional ends equal, straight.

Middle pupal stage: Long triangle, narrowing towards metacoxal.

Late pupal stage: Almost the same as the last stage.

Adult: Almost the same as the last stage.

Developmental changes: The original site changes from the metasternal discrimen to the latero-basal area of metafurca in the middle pupal stage. The insertional end becomes narrower in the middle pupal stage.

IIIscm2 M. netafurca-coxalis posterior
Initial pupal stage: Absent.
Middle pupal stage: Absent.
Late pupal stage: Absent.
Adult: O: ventro-lateral area of metafurca. I: ventro-lateral margin of metacoxal rim. Broad medially and narrowing towards both ends, slightly bent posterad.
Developmental changes: Not applicable.

IIIscm3 M. metafurca-coxalis medialis
Initial pupal stage: Absent.
Middle pupal stage: O: latero-ventral area of metafurca. I: proximo-ventral margin of metacoxal rim. Approximate parallelogram, original end narrower than insertional end, straight.
Late pupal stage: Original end broader than insertional end.
Adult: Almost the same as the last stage.
Developmental changes: The original end becomes broader in the late pupal stage. The insertional end becomes narrower in the late pupal stage.

IIIscm4 M. metafurca-coxalis lateralis
Initial pupal stage: Absent.
Middle pupal stage: O: latero-median area of metafurca. I: latero-median margin of metacoxal rim. Long triangle, narrowing towards metacoxa, slightly bent anterad.
Late pupal stage: Straight.
Adult: Almost the same as the last stage.
Developmental changes: The muscle is slightly bent anterad in the middle pupal stage, and straight in the late pupal stage and adult.

IIIscm6 M. metafurca-trochanteralis
Initial pupal stage: Absent.
Middle pupal stage: O: latero-median area of metafurca. I: metatrochanter. Long triangle, narrowing towards metatrochanter, curved.
Late pupal stage: Straight.
Adult: Almost the same as the last stage.
Developmental changes: The muscle is curved in the middle pupal stage, and straight in the late pupal stage and adult.
