# Supplementary material for: Transformation of Internal Thoracic Structures of Callobruchus maculatus (Coleoptera: Bruchidae) from Larva to Adult
Source: Insects. 2025 Mar 19;16(3):324. doi: 10.3390/insects16030324 (PMC11943184; doi:10.3390/insects16030324)
Supplement: Supplementary file 1 [file insects-16-00324-s001.zip › S5 Thoracic measurements of pupae and adult.pdf]

Supplementary Material S5: Thoracic length, width and height, muscular absolute and relative volumes of the pupae and adult

Table S1 The absolute volumes (μm^3) of thoracic muscles in each pupal developmental stage. The absolute width longer than that in the last developmental stage is denoted in blue, while shorter length is denoted in yellow. Absent is denoted with "-".

	Initial pupa	Middle pupa	Late pupa	Adult
		Prothorax		
**Idlm1**	-	139737.88	708833.08	2058218.75
**Idlm2**	-	160646.94	915967.69	2385952.47
**Idlm3**	-	-	141595.17	497860.98
**Idlm5**	-	270199.96	400829.14	8956988.53
**Idlm6**	-	-	168291.60	-
**Idvm2**	-	-	669132.86	2174150.21
**Idvm6**	-	-	525134.40	770153.69
**Idvm7**	-	293042.77	1921346.98	5787767.54
**Idvm8**	-	-	262234.26	555438.29
**Idvm9**	-	-	-	93311.81
**Idvm10**	-	-	-	217617.11
**Idvm18**	-	156124.61	1174933.77	2575706.28
**Itpm3**	-	21317.38	86434.29	619504.48
**Itpm4**	-	-	-	481376.05
**Itpm5**	-	379475.63	1373764.01	327859.39
**Itpm6**	-	266378.70	2136097.11	1682195.48
**Ipcm6**	-	324129.02	1671634.33	4401124.18
**Ivlm1**	-	-	41016.68	115600.17
**Ivlm3**	-	-	182458.77	399396.92
**Ivlm7**	-	183859.55	1029787.24	2069094.46
**Iscm4**	-	-	50614.53	163147.15
		Mesothorax		
**IIdlm1**	-	121764.10	759019.00	1566068.66
**IIdlm2**	-	148589.95	852908.08	1370602.82
**IIdvm1**	-	207988.95	681524.35	3049313.96
**IIdvm2**	-	-	388958.11	1166865.95
**IItpm1**	-	-	234144.84	692584.26
**IItpm4**	-	-	213625.02	552422.34
**IIspm2**	-	-	290890.06	996007.56
**IIspm7**	-	-	209009.78	783908.27
**IIpcm4**	-	139267.93	994319.56	1693185.43
**IIvlm3**	-	268551.27	863202.44	678612.62
**IIscm1**	-	-	117240.80	171966.53

**IIscm2**	-	102873.52	359115.97	756090.66
**IIscm6**	-	167341.85	1486014.54	2026596.92
**Metathorax**				
**IIIdlm1**	-	732549.14	3343291.62	11007243.60
**IIIdlm2**	-	434937.81	2114008.26	7466500.38
**IIIdvm1**	477710.87	1722956.19	7139450.52	33407179.90
**IIIdvm2**	664123.41	2446275.76	11211888.40	43688728.90
**IIIdvm4**	161387.69	1890536.87	6437789.04	21801581.30
**IIIdvm5**	-	326671.39	1978092.20	3158162.50
**IIIdvm8**	-	523866.82	1596497.04	3068312.18
**IIItpm1**	-	91571.53	163967.21	282437.29
**IIItpm3**	-	37496.10	94072.77	273160.94
**IIItpm7**	-	87534.56	74448.45	228150.11
**IIItpm8**	-	-	-	208055.17
**IIItpm9**	-	93798.03	112388.30	672009.50
**IIItpm10**	-	-	-	288960.43
**IIIspm1**	281092.41	1600783.78	8046968.41	28155306.40
**IIIpcm3**	281092.41	1600783.78	8046968.41	28155306.40
**IIIpcm4**	149855.15	796324.10	3222162.69	11383587.80
**IIIscm1**	135865.35	348859.34	1998122.18	4024849.58
**IIIscm2**	234012.52	222426.53	751602.479	1552542.56
**IIIscm3**	-	-	-	949443.05
**IIIscm4**	-	270770.07	2054829.13	4086459.59
**IIIscm6**	-	144244.81	875494.43	1793682.98

Table 2. The relative volumes (= absolute volume * 10^9 / (length * width * height)) of thoracic muscles in each pupal developmental stage. The relative width longer than that in the last developmental stage is denoted in blue, while shorter length is denoted in yellow. Absent is denoted with "-".

	Initial pupa	Middle pupa	Late pupa	Adult
**Prothorax**				
**Idlm1**	-	29.46	162.81	340.81
**Idlm2**	-	33.87	210.38	395.08
**Idlm3**	-	-	32.52	82.44
**Idlm5**	-	56.97	92.06	1483.13
**Idlm6**	-	-	38.65	-
**Idvm2**	-	-	153.69	360.00
**Idvm6**	-	-	120.61	127.52
**Idvm7**	-	61.79	441.30	958.36
**Idvm8**	-	-	60.23	91.97
**Idvm9**	-	-	-	15.45

**Idvm10**	-	-	-	36.04
**Idvm18**	-	32.92	269.86	426.50
**Itpm3**	-	4.49	19.85	102.58
**Itpm4**	-	-	-	79.71
**Itpm5**	-	80.01	315.53	54.29
**Itpm6**	-	56.17	490.62	278.54
**Ipcm6**	-	68.34	383.94	728.75
**Ivlm1**	-	-	9.42	19.14
**Ivlm3**	-	-	41.91	66.13
**Ivlm7**	-	38.77	236.52	342.61
**Iscm4**	-	-	11.63	27.01
**Mesothorax**				
**IIdlm1**	-	25.67	174.33	259.32
**IIdlm2**	-	31.33	195.90	226.95
**IIdvm1**	-	43.86	156.53	504.92
**IIdvm2**	-	-	89.34	193.21
**IItpm1**	-	-	53.78	114.68
**IItpm4**	-	-	49.07	91.47
**IIspm2**	-	-	66.81	164.92
**IIspm7**	-	-	48.01	129.80
**IIpcm4**	-	29.37	228.38	280.36
**IIvlm3**	-	56.63	198.26	112.37
**IIscm1**	-	-	26.93	28.47
**IIscm2**	-	21.69	82.48	125.20
**IIscm6**	-	35.28	341.31	335.57
**Metathorax**				
**IIIdlm1**	-	154.46	767.89	1822.62
**IIIdlm2**	-	91.71	485.55	1236.33
**IIIdvm1**	387.54	363.29	1639.79	5531.69
**IIIdvm2**	538.77	515.81	2575.15	7234.15
**IIIdvm4**	130.93	398.63	1478.63	3609.99
**IIIdvm5**	-	68.88	454.33	522.94
**IIIdvm8**	-	110.46	366.68	508.06
**IIItpm1**	-	19.31	37.66	46.77
**IIItpm3**	-	7.91	21.61	45.23
**IIItpm7**	-	18.46	17.10	37.78
**IIItpm8**	-	-	-	34.45
**IIItpm9**	-	19.78	25.81	111.27
**IIItpm10**	-	-	-	47.85
**IIIspm1**	228.04	337.53	1848.23	4662.06
**IIIpcm3**	121.57	167.91	740.07	1884.94
**IIIpcm4**	110.22	73.56	458.93	666.45
**IIIscm1**	189.84	46.90	172.63	257.08

**IIIscm2**	-	-	-	157.21
**IIIscm3**	-	57.09	471.95	676.65
**IIIscm4**	-	30.41	201.08	297.00
**IIIscm6**	-	125.16	1565.66	2456.28
